# A draft genome of the medicinal plant *Cremastra appendiculata* (D. Don) provides insights into the colchicine biosynthetic pathway

**DOI:** 10.1038/s42003-022-04229-4

**Published:** 2022-11-25

**Authors:** Jing Wang, Jingjing Xie, Haixia Chen, Xia Qiu, Hai Cui, Yijiang Liu, Sunil Kumar Sahu, Dongming Fang, Tengyan Li, Mei Wang, Yewen Chen, Huan Liu, Jianyong Zhang, Binbin Wang

**Affiliations:** 1 Department of Medical Genetics and Developmental Biology, School of Basic Medical Sciences, Capital Medical University, Beijing, 10069 China; 2 Department of Rheumatism, Shenzhen Traditional Chinese Medicine Hospital, Shenzhen, Guangdong China; 3 The Fourth Clinical Medical College of Guangzhou University of Chinese Medicine, Shenzhen, Guangdong China; 4 State Key Laboratory of Agricultural Genomics, BGI-Shenzhen, Shenzhen, 518120 China; 5 College of Life Sciences, University of Chinese Academy of Sciences, Beijing, 100049 China; 6 School of Traditional Chinese Medicine, Capital Medical University, Beijing, 10069 China; 7 Center for Genetics, National Research Institute for Family Planning, Beijing, China; 8 Department of Biology, University of Copenhagen, Copenhagen, Denmark

**Keywords:** Plant genetics, Plant cell biology

## Abstract

*Cremastra appendiculata* (D. Don) Makino is a rare terrestrial orchid with a high market value as an ornamental and Chinese traditional medicinal herb with a wide range of pharmacological properties. The pseudobulbs of *C. appendiculata* are one of the primary sources of the famous traditional Chinese medicine “*Shancigu*”, which has been clinically used for treating many diseases, especially, as the main component to treat gout. The lack of genetic research and genome data restricts the modern development and clinical use of *C. appendiculata*. Here, we report a 2.3 Gb chromosome-level genome of *C. appendiculata*. We identify a series of candidates of 35 candidate genes responsible for colchicine biosynthesis, among which O-methyltransferase (OMT) gene exhibits an important role in colchicine biosynthesis. Co-expression analysis reveal purple and green-yellow module have close relationships with pseudobulb parts and comprise most of the colchicine pathway genes. Overall, our genome data and the candidate genes reported here set the foundation to decipher the colchicine biosynthesis pathways in medicinal plants.

## Abstract (web summary)

A draft genome assembly of *Cremastra appendiculata* (D. Don) Makino, a traditional Chinese medicine plant, identified genes responsible for plant colchicine biosynthesis.

## Introduction

*Cremastra appendiculata* (D. Don) Makino belongs to the Orchidaceae^1^ family and is widely distributed in the southern area of China^2^. As a Traditional Chinese Medicinal plant, it is generally used in China to treat a variety of diseases. In modern clinical applications, *C. appendiculata* can be used as an effective anticancer medicine^3^. For example, according to previous studies and clinical practice, *C. appendiculata* as a highly efficient and low-toxic drug, can be used to treat gastric cancer^4^, breast cancer^5^, lung cancer^6^, liver cancer^7^, and thyroid cancer^8^. Pseudobulb is the medicinal part of *C. appendiculata* which contains colchcine and has anti-tumor^3^, detumescence, heat-clearing, and detoxification effects^9^. Moreover, *C. appendiculata* contains many pharmacological effects, including regulating blood sugar level antioxidation, reducing blood press, anti-angiogenic, and antibacterial activity^10–12^.

From the dry pseudobulbs of *C. appendiculata* collected from Lijiang, Yunnan, China, Zhu Cong et al. found that the main effective ingredients are colchicine, β-colchicine, and other alkaloids. They also found that these ingredients are mainly concentrated in the outer layer of the pseudobulbs’ skin, and the content decreases from the outside to the inside^13–15^. A recent study also found colchicine in the dried pseudobulbs from both in vitro–raised and field-raised *C. appendiculata*^16^. Colchicine is an alkaloid extracted from the carmus and seeds of *Colchicum autumnale* in *Liliaceae* family^17^, which is often used clinically to treat acute gout attacks and to relieve symptoms, such as redness, swelling, heat, and pain within a few hours. As the first-line medication for gout treatment, it can identify gout and other arthritis, and prevent gout attacks^18,19^. The pseudobulbs of *C. appendiculata* are frequently found in traditional Chinese medicine formulas or preparations for gout treatment. Yet another commercial prescription *Tongfengtai*, which uses pseudobulbs as the chief ingredient, has also achieved good clinical effects. Wei-Feng Sun et al. formulated their own clinical prescription *Xiezhuo Chubi* decoction by using pseudobulbs as the main component for treating hyperuricemia and has a good clinical application effect. The basic research found this formula promotes uric acid excretion and reduces uric acid levels in model mice by upregulating miR-34a and inhibiting URAT1 mRNA expression. In recent years, the availability of genomic and transcriptomic information on a wide variety of medicinal plants has allowed us to obtain detailed insights into their metabolic pathways, defense responses, phylogeny, and evolution^20–25^.

In this study, we assembled the chromosome-level genome of *C. appendicalata* with a 2.3 Gb size and constructed the biosynthesis pathway of colchicine. We identified 35 genes of the colchicine biosynthesis pathway in *C. appendicalata* and compared their expression level in pseudobulbs, leaves, and stems from 2-years-old, 4-years-old, and 6-years-old *C. appendiculata*.

## Results

### *De novo* genome assembly and pseudo-chromosome construction

**Fig. 1:**
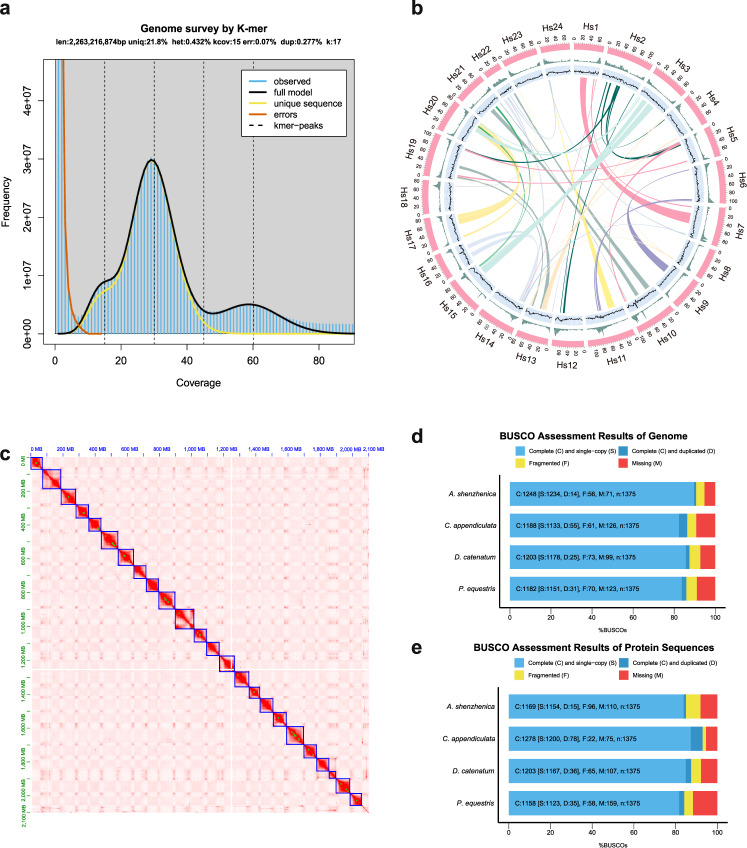
Overview of *C. appendiculata* genome assembly. **a** K-mer analysis of *C. appendiculata* genome. **b** A circular representation of the *C. appendiculata* chromosomes. The colored bands summarize the density of chromosome length (Mb) (pink), gene numbers (green), GC content (black line), and collinearity individually. **c** Hi–C interaction heat map for the *C. appendiculata* genome. The blue box represents a chromosome. **d** BUSCO assessment of *C. appendiculata, A. shenzhenica, D. catenatum*, and *P. equetris* genome. **e** BUSCO assessment of *C. appendiculata, A. shenzhenica, D. catenatum,* and *P. equetris* protein sequences.

By adopting the whole genome sequence strategy, we generated 238 Gb clean data, which represented ~100-fold coverage of the predicted 2.2632 Gb genome size by 17-mer estimation. We did not observe a significant secondary peak indicating considerable heterozygosity in the k-mer distribution (Fig. 1a). By integrating WGS and PacBio sequencing data, we assembled the draft genome of *C. appendiculata* of about 2.3597 Gb with an N50 length of 1.15 Mb, which is almost same as the previous estimates by K-mer. The largest scaffold is 11.72 Mb among them. We additionally used 250 Gb of Hi–C data to reconstruct physical maps by reordering and clustering the assembled scaffolds. We anchored 87.21% of the assembly (2.058 Gb) onto 24 pseudochromosomes using a hierarchical clustering strategy (Fig. 1b). The length of the pseudochromosomes ranged from 42.81 Mb to 117.12 Mb (Fig. 1c) with an N50 value of 80.16 Mb (Table 1). The completeness evaluation of the *C. appendiculata* genome and protein sequences with the other three species by BUSCOs (benchmarking universal single-copy ortholog) are individually presented in Fig. 1d, e, and Supplementary Data 1. The protein sequences of *Apostasia shenzhenica*, *Dendrobium catenatum*, and *Phalaenopsis equestris* are presented in Supplementary Data 2–4, respectively.

**Table 1:** Statistics of the *de novo* genome assembly of *C. appendiculata*.

Statistical level	Scaffolds	Contigs
Total number (>)	8767	16,208
Total length (bp)	2,365,912,979	2,362,341,580
Average length (bp)	269,865.75	145,751.58
N50 Length (bp)	80,155,791	677,859
N90 Length (bp)	146,279	60,438
Maximum length (bp)	117,118,055	11,563,929
Minimum length (bp)	1000	1
GC content (%)	39.39	39.39

### Protein-coding gene prediction and functional annotation

Repeat analysis showed 59.15% of the assembled *C. appendiculata* genome comprised of repetitive elements, the majority being long terminal repeats (LTRs), accounting for 56.25% of the genome. The type of DNA class repeats elements accounted for 2.7% of *C. appendiculata* genome; LINE and SINE classes represented 1.37% and 0.00046% of this genome, respectively (Supplementary Data 5). The combination of *de novo*, homology-based, and transcriptome-based predictions yielded 20,991 genes with 5.13 exons per gene on average. The average length of mRNA, exon and intron was 11,547, 270, and 2461 bp, respectively (Supplementary Table 1). The completeness assessment of the gene set of *C. appendiculata* reached 96.1% which was higher than the other three published Orchidaceae species genomes (Fig. 1e).

The prediction of gene models by various techniques was also summarized, with a large range in the number of predicted genes. A total of 99.79% of gene models have a homolog match or conserved motif in at least one of the public protein databases, including NCBI non-redundant (NR) protein databases, 99.75%, Swissprot, 81.81%; InterPro, and 84.58%; the Kyoto Encyclopedia of Genes and Genomes (KEGG), 79.58%. In addition to protein-coding genes, we also identified 97 microRNA, 430 tRNA, 2926 rRNA, and 728 small nuclear RNA genes in *C. appendiculata* genome (Supplementary Table 2).

### Comparative genomic analysis

**Fig. 2:**
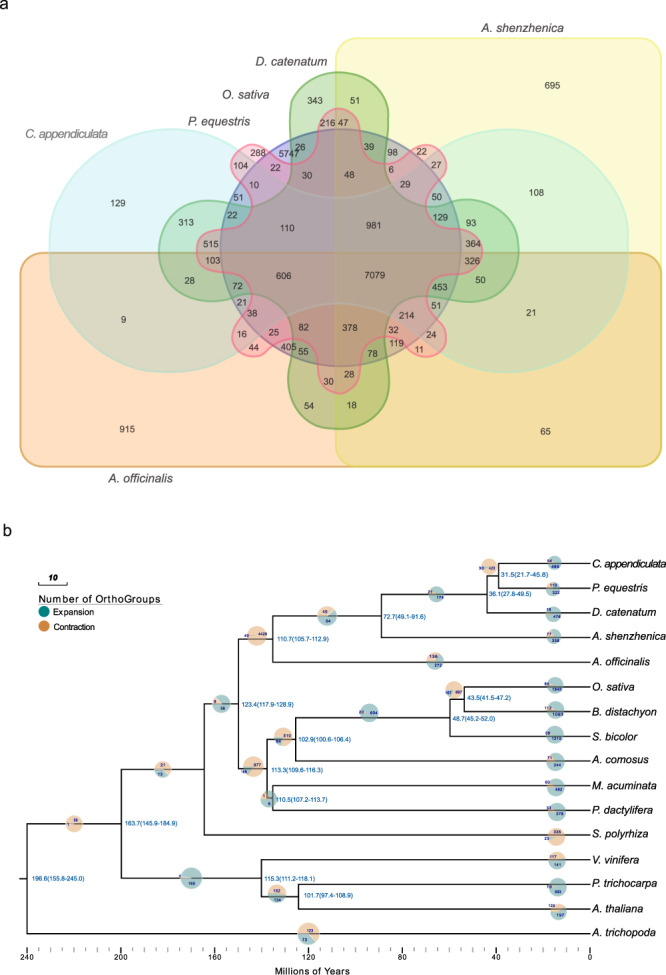
*C. appendiculata* genome evolution and the distribution of gene families among six monocot genomes. *C. appendiculata* genome evolution. **a** Shared orthologous gene clusters among *C. appendiculata*, *P. equestris*, *O. sativa*, *D. catenatum*, *A. shenzhenica*, and *A. officinalis*. **b** Phylogenetic tree and number of gene families displaying expansion and contraction of 16 plant species.

We compared our assembly with 15 other sequenced genomes from three Orchidaceae (*P. equestris, A. shenzhenica*, and *D. catenatum*), three rosids species *(Arabidopsis thaliana, Populus trichocarpa, Vitis vinifera)*, four commelinids species (*Ananas comosus, Phoenix dactylifera, Brachypodium distachyon, Musa acuminate*), two cereal crops *Oryza sativa* and *Sorghum bicolor*, a special species *Amborella trichopoda* and other monocots species *(Spirodela polyrhiza, Asparagus officinalis)*. Based on the analysis of gene family clustering, we identified 12,146 gene families in *C. appendiculata* genome. A total of 489 gene families had expanded and 54 gene families had contracted in *C. appendiculata*, 94 gene families appeared to be unique to *C. appendiculata*, respectively (Supplementary Table 3). And 7079 orthologous gene families shared by the six plants were analyzed (Fig. 2a). Geno ontology (GO) studies based on the 489 expanded gene families showed enrichment of genes encoding 81 GO terms, the most enriched five terms including metabolic process (1447), organic substance metabolic process (1336), cellular process (1335), primary metabolic process (1334) and cellular metabolic process (1312). Oxidoreductase activity, acting on NAD(P)H (21), hydrolase activity (969), hydrolase activity, acting on ester bonds (857), and O-methyltransferase activity (11) are related to colchicine biosynthesis, which was enriched by the expanded gene families (Supplementary Data 6). It is noteworthy that the unique families are also enriched in hydrolase activity (209), methyltransferase activity (11), and O-methyltransferase (8) (Supplementary Data 7). We performed KEGG enrichment analysis and found that these expanded gene families were enriched in 32 categories, such as Metabolic pathways (2554), Biosynthesis of secondary metabolites (996) and related with colchicine biosynthesis pathways, including Phenylalanine metabolism (63), and Isoquinoline alkaloid biosynthesis pathways (37). (Supplementary Data 6). Interestingly, the unique genes also enriched in phenylalanine, tyrosine, and tryptophan biosynthesis (17), tyrosine metabolism (16), and phenylalanine metabolism pathways (14) which are related to the colchicine biosynthesis (Supplementary Data 7).

We estimated the divergence times of 16 plants based on 2430 single-copy orthogroups by constructing a phylogenetic tree. These gene families of the 16 plant species were compared with their most recent common ancestor (MRCA). As expected, as a member of the Orchidaceae, *C. appendiculata* is a sister to all other orchids. The most closely related one is *P. equestris* which is separated approximately 31.5 million years ago. Although the evolutionary distance from orchid to dicotyledon such as *V. vinifera, P. trichocarpa, A. thaliana,* and a single species from Amborellaceae—*A. trichopoda* was relatively large (Fig. 2b).

### Genome evolution

**Fig. 3:**
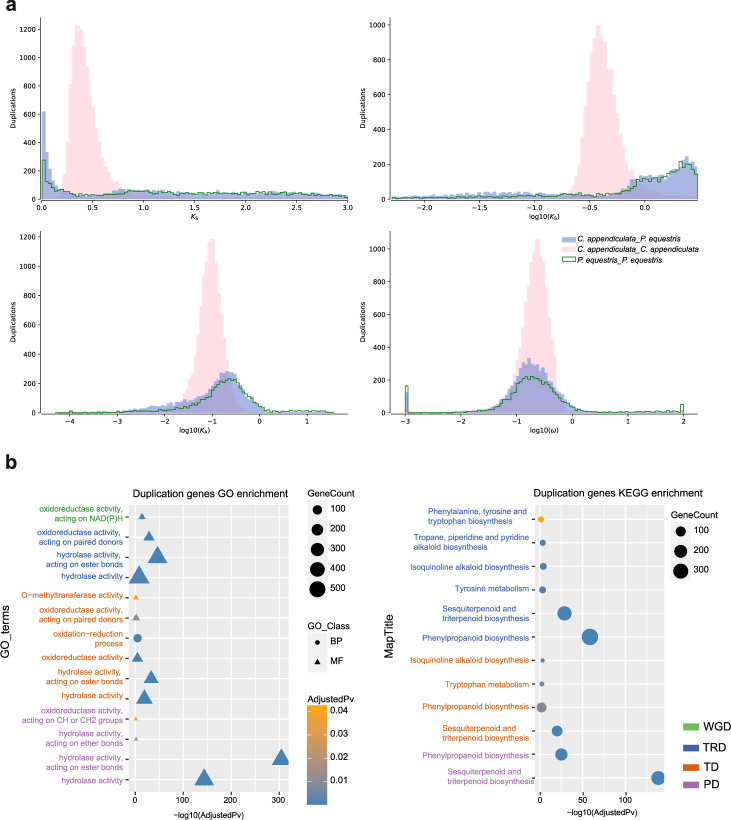
The analysis of *C. appendiculata* WGD event. **a** The distribution of synonymous substitution rate (*K*_s_) distance values observed for *C. appendiculata* paralogs, *P. equestris* paralogs, and *C. appendiculata*–*P. equestris* orthologs, respectively. **b** The GO and KEGG function enrichment analysis of different duplication genes: genome duplication (WGD), tandem duplication (TD), proximal duplication (PD), and transposed duplication (TRD).

Ancient whole-genome duplications (WGDs or polyploidy) are prevalent in plants, and may have contributed to plant adaptation^26^. In this study, we compared *C. appendiculata* and *P. equestris* employing synonymous substitution per synonymous site (Ks) approach to determine whether *C. appendiculata* genome had undergone WGD. There was a peak between *K*_s_ values of 0.66, indicating the *C. appendiculata* genome had undergone an ancient WGD event (Fig. 3a). We detected 61 syntenic blocks across the whole genome, including 20,991 genes. However, the average number of syntenic gene pairs per block was only 11, which likely cause difficulty in identifying the WGD event. Only 25 syntenic blocks were found in the *P. equestris* genome, causing their own WGD events undetectable (Supplementary Table 4). Furthermore, we assessed the intergenomic collinearity between *C. appendiculata* and *P. equestris* genomes. We found a Ks peak at 0.3462 indicating a common WGD event between different representative genomes dated 33.93 million years ago which is earlier than the divergence time (Supplementary Table 5). In addition to the WGD, tandem duplication (TD), proximal duplication (PD), transposed duplication (TRD), and dispersed duplication (DSD) belong to single gene duplication^27–29^ are also more frequently associated with the evolution of species^30^. We identified the different modes of duplicated gene pairs in *C. appendiculata*, including 760 WGD pairs, 852 TD pairs, 739 PD pairs, 3999 TRD pairs, and 80,616 DSD pairs. We also investigated the duplicated genes’ function, WGD genes were predominant in the ribosome (90), photosynthesis (85), oxidative phosphorylation (57), RNA polymerase (22), and flavonoid biosynthesis pathways (11) (Supplementary Data 8). A part of TD, PD, and TRD genes that contributed to hydrolase activity, oxidoreductase activity, and Phenylpropanoid biosynthesis pathway. Many TD and TRD genes are enriched in phenylpropanoid biosynthesis, sesquiterpenoid, and triterpenoid biosynthesis, Tyrosine metabolism, Isoquinoline alkaloid biosynthesis, Phenylalanine, tyrosine and tryptophan biosynthesis pathways which closely related to the colchicine biosynthesis. Especially, only six tandem duplication genes are enriched in O-methyltransferase activity terms (Fig. 3b). Therefore, the different duplicated modes have different functions for the species’ evolutionary process. Tandem duplication and transposed duplication may play an important role in colchicine synthesis.

### Candidate genes of colchicine biosynthetic pathway and expression analysis

**Fig. 4:**
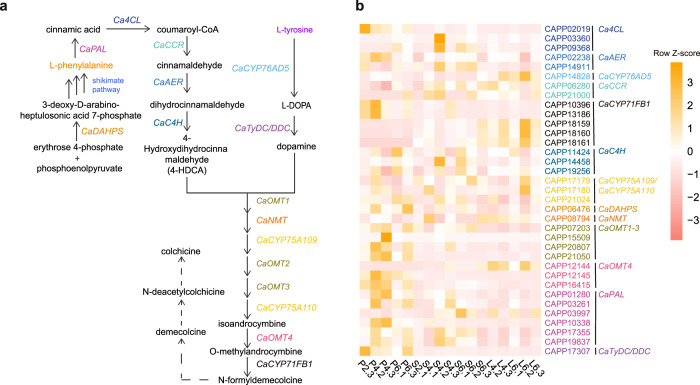
Biosynthesis pathway of colchicine and the expression level of candidate enzymes in the pathway. **a** The colchicine biosynthesis pathway. **b** The expression level of colchicine-related genes in *C. appendiculata* genome. “P”: Pseudobulb, “S”: Stem, “L”: Leaf. 2: Two years old, 4: Four years, 6: Six years. “.1, .2, .3” means sample duplications. Row *Z*-score means the normalization of FPKM values by the *z*-score method of each row. Yellow color represents a high expression level, and pink color represents a low expression level.

The colchicine core is constructed from the amino acids l-phenylalanine and l-tyrosine^31–39^ (Fig. 4a). Phenylalanine and tyrosine are the precursor of 4-hydroxydihydrocinnamaldehyde (4-HDCA) and dopamine^40,41^, respectively. A recent study elucidated eight genes in the colchicine biosynthesis pathway and engineered the whole pathway using 16 genes in *N. benthamiana*^42^. A total of six enzymes are involved in the conversion of phenylalanine to 4-HDCA, including phenylalanine ammonia-lyase (PAL), 4-coumarate: CoA ligase (4CL), cinnamoyl-CoA reductase (CCR), alkenal reductase-like protein (AER), cytochrome P450 family 73 subfamilies A polypeptide 253 (C4H) and 3-deoxy-d-arabino-heptulosonate-7-phosphate-synthase (DAHPS). From L-tyrosine to dopamine reactions contained two enzymes, TyDC/DDC and CYP76AD5. In addition, a total of eight functionally validated enzymes (GsOMT1, GsNMTt, GsCYP75A109, GsOMT2, GsOMT3, GsCYP75A110, GsOMT4, and GsCYP71FB1) in colchicine sourced *Gloriosa superba* L^42^ were download from NCBI as the reference sequences to perform a BLAST search with the predicted *C. appendiculata* protein sequences. We obtained 35 homologous genes that are potentially involved in the colchicine biosynthetic pathway of *C. appendiculata* (Supplementary Table 6) and compared those candidate genes with their homologs in *P. equestris, A. shenzhenica, D. catenatum*, using *A. trichopoda* as an outgroup. We identified gene loss and duplication events along the lineage leading to *C. appendiculata* by manually examining each gene tree individually. Especially, we discovered one gene duplication event in the CaAER, CaCYP76AD5, and CaCCR gene families, respectively. Three gene duplications in both CaOMT1-3 and CaCYP71FB1 gene families, and four gene duplications in the CaPAL gene families (Supplementary Data 9). Specially, we found two duplications from CaCYP71FB1 gene families that were produced from the tandem duplication events. There is no gene loss event identified in these gene families.

Moreover, we compared the 35 candidate gene expression level in different tissues of 2-years-old, 4-years-old, and 6-years-old *C. appendiculata*. However, we aligned those RNA reads to the assembled genome of *C. appendiculata* using Hisat2 and found four two-years-old samples have low mapping rates (<60%) (Supplementary Table 7), so we excluded these samples in the subsequent analysis. Interestingly, the *CaTyDC/DDC* genes were highly expressed in pseudobulbs, and *CaCYP76AD5* expression was high in leaves. *CaDAHPS* and *CaPAL* genes were expressed higher in pseudobulbs and stem rather than leaves (Fig. 4b).

### O-methyltransferases evolution in *C. appendiculata*

**Fig. 5:**
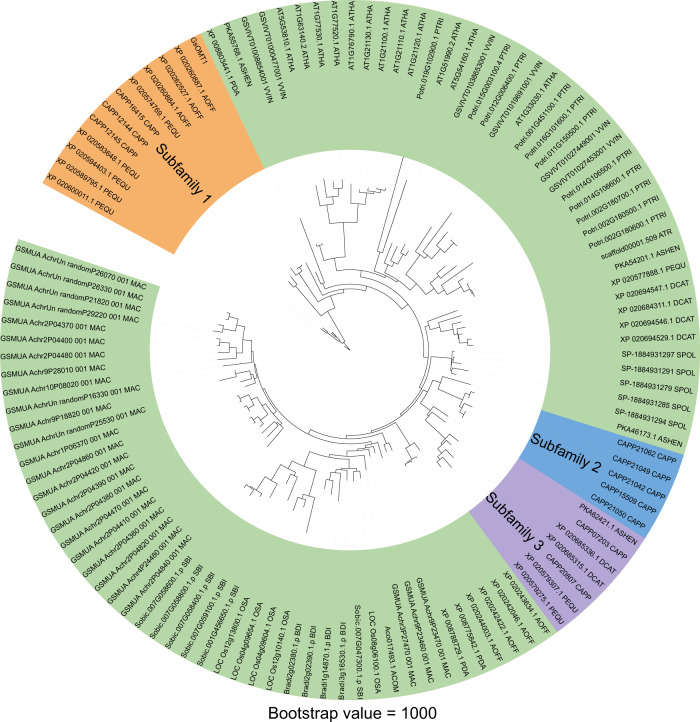
The phylogenetic tree of OMTs. Subfamily1: yellow, subfamily 2: blue, subfamily 3: purple. VVIN *Vitis vinifera*, PEQU *Phalaenopsis equestris*, ASHEN *Apostasia shenzhenica*, SPOL *Spirodela polyrhiza*, ACOM *Ananas comosus*, DCAT *Dendrobium catenatum*, PDA *Phoenix dactylifera*, OSA *Oryza sativa*, ATR *Amborella trichopoda*, AOFF *Asparagus officinalis*, BDI *Brachypodium distachyon*, MAC *Musa acuminata*, ATHA *Arabidopsis thaliana*, CAPP *Cremastra appendiculata*, SBI *Sorghum bicolor*, PTRI *Populus trichocarpa*.

O-methyltransferase (OMT) plays an important role in colchicine biosynthesis. We identified ten *CaOMTs* and construct a phylogeny tree-based classification of genes that manifest *CaOMTs* were divided into three distinct subfamilies. Since the results of *CaOMT1*, *CaOMT2*, *CaOMT3* obtained by sequence alignments are consistent, we collectively refer to them as CaOMT1-3. Subfamily 1 contained three *CaOMT4s* (CAPP16415, CAPP12144, CAPP12145) from *C. appendiculata*, as well as including OMTs from *A. officinalis* (_AOFF) and *P. equestris* (_PEQU). Subfamily 2 only contains five *CaOMT*s (CAPP21062, CAPP21049, CAPP21042, CAPP15509, CAPP21050). Subfamily 3 contains two *CaOMT1-3s* (CAPP07203, CAPP20807) and other candidate genes from *A. shenzhenica*, *D. catenatum* (_DCAT), and *P. equestris* (Fig. 5). Interestingly, CAPP07203, CAPP15509, CAPP21050 (*CaOMT1-3*) and CAPP12144, CAPP12145 (*CaOMT4*) are in one scaffold.

### Differentially expressed gene analysis and co-expression reveal the active biosynthesis sites

**Fig. 6:**
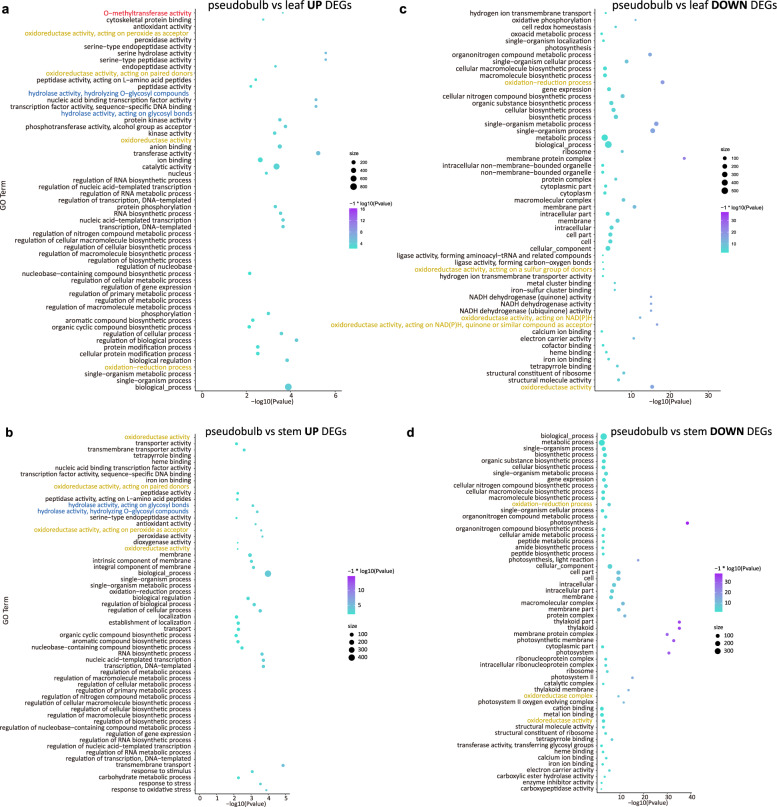
The GO enrichment analysis of DEGs between pseudobulb vs leaf and pseudobulb vs stem. **a**, **c** Up- and downregulated DEGs between pseudobulb and leaf, respectively. **b**, **d** Up- and downregulated DEGs between pseudobulb and stem, respectively.

To investigate the different expression levels of colchicine-related genes of *C. appendiculata*, we compared the expression values of pseudobulb, leaf, and stem in pairs. GO enrichment analysis of those DEGs showed each group of DEGs (pseudobulb vs leaf, pseudobulb vs. stem, and leaf vs stem) was enriched in several oxidoreductase activities or oxidoreductase activity-related GO terms. However, there’s only one O-methyltransferase activity GO terms (10) enriched in the up-regulated DEGs between pseudobulb and leaf (Fig. 6a). It contained ten genes (CAPP06033, CAPP06032, CAPP12145, CAPP06524, CAPP16938, CAPP20807, CAPP16415, CAPP06696, CAPP06686, CAPP06688). Additionally, hydrolase activity, acting on glycosyl bonds (25) and hydrolase activity, hydrolyzing O-glycosyl compounds (24) GO terms enriched in up-regulated genes between pseudobulb and stem (Fig. 6b). Several oxidoreductase activity terms were enriched of down-regulated genes between pseudobulb-vs.-leaf and pseudobulb-vs.-stem DEGs (Fig. 6c, d and Supplementary Data 10).

**Fig. 7:**
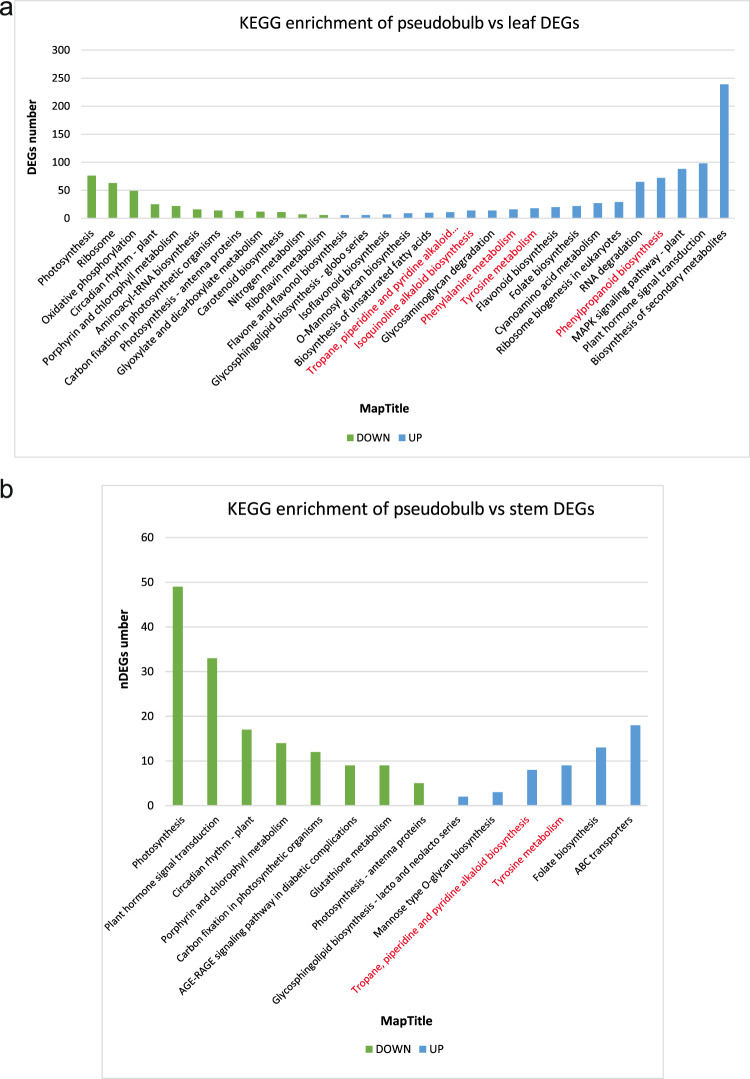
The KEGG enrichment analysis of DEGs between pseudobulb vs. leaf and pseudobulb vs. stem. **a** KEGG enrichment analysis of DEGs between pseudobulb and leaf. **b** KEGG enrichment analysis of DEGs between pseudobulb and stem. Down: green. Up: blue.

Interestingly, the KEGG enrichment results show a consistent pattern. The up-regulated DEGs between pseudobulb and leaf are enriched in Tyrosine metabolism (18), Isoquinoline alkaloid biosynthesis (14), Tropane, piperidine, and pyridine alkaloid biosynthesis (11), Phenylpropanoid biosynthesis (72) and phenylalanine metabolism (16) pathways (Fig. 7a). Besides, the KEGG enrichment analysis results of up-regulated DEGs between pseudobulb and stem also enriched in Tropane, piperidine, and pyridine alkaloid biosynthesis (8) and Tyrosine metabolism pathways (9) (Fig. 7b, Supplementary Data 11).

By manually searching the genes of the co-expression module for whether they contained candidate colchicine genes, we screened genes with an average FPKM value of more than 1 as input files and found two modules have a high correlation with pseudobulb samples almost include all necessary enzymes in the colchicine biosynthesis pathway (Supplementary Fig. 1). The purple module incorporated *Ca4CL, CaPAL, CaAER, CaCCR, CaOMT1-CaOMT4, CaCYP71FB1*, and *CaTyDC/DDC* and the green-yellow module contained *CaCCR*, *CaNMT*, and *CaOMT4*. Besides, we also identified three modules (maroon, thistle2, and dark-turquoise) that were highly related to stem samples. However, they only included a part of coding genes which was repeated with a purple module. In addition, two *CaOMTs* (CAPP12145 and CAPP16699) individually belong to two modules (purple, green-yellow), which showed a positive and high correlation with the pseudobulb of *C. appendiculata*. Therefore, we speculated that the pseudobulb is the active site of colchicine biosynthesis.

### qRT-PCR validation of colchicine-related genes

To justify our experimental conclusions, we selected ten genes that are involved in the colchicine biosynthesis pathway to perform qRT-PCR validation tests. The expression pattern of some genes was in accordance with each other and moderately correlated, such as *CaTyDC/DDC*, *CaPAL, Ca4CL*, and *CaOMT1-3* (Supplementary Fig. 2a–f). Some genes have different expression patterns (Supplementary Fig. 2g–j). The correlation coefficient of log2 Fold Change values between qRT-PCR and RNA-seq data was −0.9 in pseudobulb vs. leaf and 0.69 in pseudobulb vs. stem, respectively (Supplementary Fig. 2k, l). Overall, these observations suggest the reliability of the present RNA-Seq data. The original CT values and related data were summarized in Supplementary Data 12.

## Discussion

*Cremastra appendiculata* is a rare terrestrial orchid with a high market value as an ornamental and Chinese traditional medicinal herb with a wide range of pharmacological properties. Our study combined Pacbio and Hi–C technology to provide a chromosome-level genome of *C. appendiculata* with 2.3 Gb of Orchidaceae family.

Polyploidization is a common phenomenon in angiosperms because it helps plants adapt to their surroundings and evolve their genomes^43,44^. The most recent common ancestor of orchids experienced one polyploidization event^45–47^. Our comparative analysis showed that *C. appendiculata* also experienced one WGD event, and that led to the expansion of several gene families, which were enriched in Phenylalanine metabolism, Flavone and flavonol biosynthesis, Flavonoid biosynthesis and Isoquinoline alkaloid biosynthesis pathways. These KEGG pathways are involved in colchicine biosynthesis. The TD duplicated genes also exhibited close relationships with the colchicine biosynthesis pathway. *C. appendiculata* is particularly known for its anti-tumor effect. Previous research found the total alkaloids of *C. appendiculata* can inhibit the proliferation of esophageal cancer cells, this is may be related to the inhibiting effect of colchicine^3^. Colchicine is the oldest plant natural product still used to treat a variety of ailments in humans, including gout and other articular inflammation disorders^42^. We constructed a colchicine biosynthetic pathway in *C. appendiculata* genome and identified 35 candidate genes. The Shikimate pathway is highly conserved in plants which is the central intermediate to a large range of secondary metabolites in plants, such as alkaloids, flavonoids, and lignins^48,49^. As the first enzyme, DAHPS is encoded by three genes in the *Arabidopsis* genome, only a single gene and two to eight isoforms are found in algae and other higher plant species. However, we only found a single *DAHPS* gene in *C. appendiculat*a genome^50^. Likewise, PAL is a speed-limiting step in phenylpropanoid metabolism^51^, which plays a vital role in the adaptation and resistance of plants in unseemly environmental conditions^52^. Previous studies have recognized and functionally described four PAL gene family members in *Arabidopsis thaliana*^53–55^, five in *P. trichocarpa*^56^, three in *Scytellaris baicalensis*^57^, and three in *Coffea anaphora*^58^. In our study, we identified six *PAL* genes in *C. appendiculata*. The TyDC/DDC enzyme catalyzes the synthesis of tyramine and dopamine, which are the first steps in the biosynthesis of the tetrahydroisoquinoline alkaloids. In Opium poppy, TYDC2-like transcripts predominate in stems and are also found in roots in mature plants, whereas TYDC1-like transcripts are only found in roots^59^. In yet another study, colchicine alkaloids were found to be distributed throughout the Colchicum plant, the seeds and corms contain the highest quantities^42^, therefore, our results, in accordance with earlier reports, suggest that the accumulation and concentration of colchicine vary from species to species. Previous researchers have identified that colchicine is the alkaloid component of pseudobulbs of *C. appendiculata*. The complete biosynthesis pathway of colchicine has remained unclear. Here we further experimentally validated the expression pattern of 16 representative colchicine pathway-related genes. The genomic and transcriptome data of *C. appendiculata* provided insights into the evolution of the colchicine pathway. Overall, our findings pave the way for more investigation into the functional genes involved in the production of colchicine.

## Methods

### Plant sample collection and sequencing

The *C. appendiculata* samples were cultivated in Baoshan, Yunnan Province, China. The species identity was taxonomically confirmed by Prof. Zhang Shouzhou (Fairy Lake Botanical Garden, Shenzhen & Chinese Academy of Sciences). The fresh and young leaves were collected to extract genomic DNA for PacBio, Hi–C, and WGS sequencing. SMRTbell Template Prep Kit 1.0, Sequel Binding Kit 1.0, and Sequel DNA Internal Control 1.0 were used for template preparation, DNA binding, and DNA control step, respectively. We collected the fresh leaves for the Hi-C experiment. First, we cut them into fragments with 50 ml MC buffer (10 mM Potassium Phosphate, pH 7.0, 50 mM NaCl, 0.1 M sucrose) and 1.39 ml 37% methanal to infiltrate those fragments. The methanol-processed tissues were ground to powder in liquid nitrogen to extract DNA by the CTAB method^60^. The Hi–C library was constructed on BGISEQ-500 matched manual and sequenced on the BGISEQ-500 platform.

Two-years-old, 4-years-old, and 6-years-old *C. appendiculata* whole plant was divided into leaf, stem, and pseudobulb parts for RNA-seq sequencing. Total RNA was extracted from leaves, stems, and pseudobulbs by using the βBIOZOL method. The concentration, purity, and integrity of these RNA samples were measured by Qubit 2.0, Nanodrop, and Agilent 2100 methods, respectively, to ensure they are suitable for library construction and sequencing. RNA samples with RNA integrity number (RIN) value over seven proceeded with library preparation by MGIEasy RNA kit (CAT# 1000006383). Quality validation of raw reads and clean reads were performed using FastQC (version 0.11.3)^61^. Low-quality reads were filtered using Trimmomatic (version 3)^62^. Cleaned reads were mapped to the reference genome using Hisat2 (version 2.1.0)^63^. PCA analysis result is presented in Supplementary Fig. 3.

### Genome assembly and chromosome anchoring

Whole Genome Sequencing generated 238 Gb short-reads data (100-fold coverage of the genome) for k-mer analysis and corrected base errors of the assembled genome by long reads. Jellyfish (v 2.2.6)^64^ and GenomeScope^65^ were used for k-mer frequency statistics and accurate estimation of genome size respectively. The single Molecule Real Time long-read library was constructed and sequenced on the PacBio Sequel platform (114 Gb data, 50-fold coverage of the genome). Long reads were generated for *de novo* assembly using NextDenovo (https://github.com/Nextomics/NextDenovo) and used NextPolish to fix base errors in the *C. appendiculata* genome generated by noisy long reads with a combination of both short read data and long read data (https://github.com/Nextomics/NextPolish). For Hi–C sequencing, a total of 250 Gb (105-fold coverage of the genome) data were generated on the BGISEQ-500 platform. We used juicer pipeline^66^ for generating Hi-C maps and 3D *de novo* assembly (3D-DNA) pipeline^67^ with generated Hi–C linking information to create accurate genome assemblies with chromosome-length scaffolds. Using the RNA sequencing data from three different *C. appendiculata* tissues (leaves, stems, and pseudobulbs) to map back to our assembled genome, and most of the samples reached a 90%~ mapping rate.

### Identification of repetitive sequences

By using RepeatMasker v4.0.6 and RepeatProteinMask v4.0.6^68^ identified TEs and other repeat elements in the known repeat database—Repase^69^ in the *C. appendiculata* genome. Tandem repeats were identified using Tandem Repeats Finder (TRF, 4.07b)^70^. De novo repeat libraries were constructed using the de novo prediction programs Piler v1.0^71^ and LTR-FINDER v1.06^72^, followed by RepeatMasker v.4.0.6 to get the final results. By combining these libraries as a database, repeats in this genome were identified and classified using RepeatMasker.

### Gene model prediction and functional annotation

The repeat-masked *C. appendiculata* genome sequence was used for gene predictions. MAKER-P v2.31 was used to predict protein-coding genes based on homology, RNA-seq data, and *de novo* prediction evidence. Genemark-ES v4.21^73^ was self-trained using the default criteria. By running the first round of MAKER-P analysis with default parameters and generated GeneMark HMMs. SNAP^74^ was used for training these gene models subsequently. The second and final rounds of MAKER-P with default parameters generated final gene models, which passed to functionally annotated aligning their protein sequence with KEGG^75^, COG^76^, SwissProt^77^, TrEMBL, and NCBI non-redundant (NR) protein databases with BLASTP (E-value ≤ 1e-05). For ncRNA annotation, tRNA genes were identified with tRNAscan-SE v1.3.1^78^. By aligning the assembled genome with the rRNA sequences of *A. thaliana* using BLASTN (*E*-value ≤ 1e−05) to identify the rRNA of *C. appendiculata*. For snRNA and miRNA annotation, we aligned the assembled genome with the Rfam database^79^ by BLASTN (*E*-value ≤ 1e−05).

We used the website tool iTAK^80^ to predict transcription factors (TFs), Transcriptional regulators (TRs), and protein kinases (PKs) in *C. appendiculata* genome and other species’ genomes. A total of 1,269 TFs, 297 TRs, and 664 PKs were predicted in *C. appendiculata* genome, respectively. Carbohydrate-Active enzymes (CAZymes) are involved in the synthesis, metabolism, and recognition of complex carbohydrates, i.e., disaccharides, oligosaccharides, polysaccharides, and glycoconjugates. We used CAZy database^81^ and identified 831 CAZymes in *C. appendiculata* genome. Based on an R-genes prediction pipeline, we got 12 kinds of resistance genes (Rgenes) in *C. appendiculata* genome and other species’ genomes. The Rgenes number of *C. appendiculata* is less than the close species: *A. shenzhenica, P. equestris*, and *D. catenatum*. KofamKOALA is a web server to assign KEGG Orthologs (KOs)^75^ of *C. appendiculata* protein sequences by homology search against a database of profile hidden Markov models (KOfam) with pre-computed adaptive score thresholds. The summarized data of the above annotation resulted in *C. appendiculata* and other species’ genomes are shown in Supplementary Data 13.

### WGD analysis and four other different duplication modes of identification

We used wgd software^82^ to perform the *K*_s_ distribution analysis. The DupGen_finder was developed to identify different modes of duplicated gene pairs^83^. MCScanX^84^ algorithm was incorporated into this pipeline. A whole set of potential homologous gene pairs obtained from intra-species BLASTP output were used to detect WGD-derived, TD-derived, PD-derived, and TRD-derived gene pairs successively. The remaining BLASTP hits were dispersed duplications.

### Gene family construction and divergence time estimation

Whole-genome sequences from *Phalaenopsis equestris, Apostasia shenzhenica*, *Dendrobium catenatum*, *Arabidopsis thaliana, Populus trichocarpa, Vitis vinifera, Ananas comosus, Phoenix dactylifera, Brachypodium distachyon, Musa acuminate, Oryza sativa*, *Sorghum bicolor*, *Amborella trichopoda*, *Spirodela polyrhiza* and *Asparagus officinalis* were used for gene family clustering analysis with *C. appendiculata* genome. Pairwise sequence similarities between all protein sequences were calculated using BLASTP with an E-value cutoff of 1e−5. Gene family clusters among different species were identified using OrthoMCL software (Version 1.4)^85^.

The output of OrthoMCL was passed to identify gene families. The single-copy genes in all species analyzed were aligned using MAFFT (v7.273)^86^. Each gene tree was constructed by using RAxML-ng^87^ (v 0.6.0). We used Astral v5.6.3^88^ with 100 bootstrap replicates to construct the species' phylogenetic tree. The divergence time between *C. appendiculata* and other species was estimated using MCMCTREE (https://github.com/PuttickMacroevolution/MCMCtreeR) (v4.5) with the default parameters. The expansion and contraction of gene family numbers were predicted using CAFÉ^89^ (v2.1) by employing the phylogenetic tree and gene family statistics.

### Candidate genes of colchicine biosynthetic prediction

We identified putative colchicine pathway genes by blast the reference genes from *Gloriosa superba* colchicine pathway to all predicted *C. appendiculata* protein sequences (*e*-value = 1e−10). We identified orthologs of the candidate colchicine genes with other species, including *P equestris, A. shenzhenica*, *D. catenatum*, *A. thaliana, P. trichocarpa, V. vinifera, A. comosus, P. dactylifera, B. distachyon, M. acuminate, O. sativa*, *S. bicolor*, *A. trichopoda*, *S. polyrhiza*, and *A. officinalis*. Then, we aligned the coding sequences of each gene family using MAFFT (v7.273). We used PAL2NAL (version 14.1) to convert a multiple sequence alignment of proteins and the corresponding DNA (or mRNA) sequences into a codon-based DNA alignment. TrimAL (v1.4. 15) was used to remove the poorly aligned regions from an alignment matrix to increase the quality of subsequent analyses. Before constructing the phylogeny, we removed the dicotyledon plants. A gene tree was then constructed with PhyML (v3.0) using maximum likelihood for each gene family.

### Transcriptome and co-expression analysis

Hisat2 software to map clean RNA sequencing reads to *C. appendiculata* genome with the following parameters: hisat2-align-s --wrapper basic-0 -t -x. By using the Hisat2 mapping results to generate transcriptome gtf profile and then calculated the expression level for *C. appendiculata* genes (FPKM, TPM, and expression count data) by Stringtie software^90^. The gene count data as the input file to conduct differential expression analysis by DEseq2 package^91^ in R. The screen criteria of differential expression genes are adjusted *p* value < 0.05, log2FoldChange > 1 (up-regulated), or log2FoldChange < −1 (down-regulated). Next, we performed co-expression analysis to identify a highly co-expressed gene cluster representing the colchicine biosynthetic pathway. The whole analysis was conducted using WGCNA package^92^ in R. We used all FPKM values as an input file. First, the powers value (soft thresholding powers) of 9 was selected for correlation coefficient weighting to expand the difference between genes’ correlation. Next, the function of adjacency () with default parameters to build the adjacency metrics. Then, constructing a topological overlap matrix (TOM) using Tomsimilarity () function with default parameters based on the gene expression value matrix. TOM values represent the similarity between two genes which can be used to build a cluster tree. Further, modules were derived using cutreeDynamic () function with parameters as deepSplit = 2, pamRespectsDendro = FALSE, minClusterSize = 30. Co-expression modules were built by using hclust () function with method = ‘average’. We defined cutHeight = 0.25 to merge similar modules by mergeCloseModules () function. Finally, we imported the sample trait file to associate the co-expression module. Genes within the same module have high co-expression similarity, suggesting that they participate in similar regulatory pathways or functions in similar cellular regions. The DEseq2 and WGCNA Rscript were supplied in Supplementary Data 14.

### Validation of differentially expressed genes by qRT-PCR

We used RNAprep Pure Plant Plus kit (TIANGEN, DP441) to extract total RNA and confirmed its integrity by agarose gel electrophoresis. Then, mRNA cDNA Synthesis Kit (GenePool, Cat# GPQ1803) was used to perform the inverse transcription according to the operation instruction. Finally, BIOER LineGene 9600Plus fluorescence quantitative PCR instrument was used for relative quantitative analysis of the data by the 2^−^^ΔΔCT^ method (pseudobulb as control sample). Finally, we compared the log2 fold change value between RNA-seq data and qRT-PCR to make those histograms by GraphPad software. We selected a β-tubulin gene (CAPP06738) as a reference gene. The primer list of these genes is shown in Supplementary Data 15.

### Statistics and reproducibility

Statistics analyses of differentially expressed genes among different tissues were analyzed by one-way ANOVA and followed by Tukey’s Honestly Significant Difference test for multiple groups comparison (*p* < 0.05). The statistical of qRT-PCR was performed by GraphPad Prism 8. DESeq and WGCNA analysis R scripts are provided in supplementary data 14. The number of biological replicates of qRT-PCR is three of every plant part and age.

### Reporting summary

Further information on research design is available in the Nature Portfolio Reporting Summary linked to this article.

## Supplementary information

Supplementary Information

Description of Additional Supplementary Files

Supplementary Data 1

Supplementary Data 2

Supplementary Data 3

Supplementary Data 4

Supplementary Data 5

Supplementary Data 6

Supplementary Data 7

Supplementary Data 8

Supplementary Data 9

Supplementary Data 10

Supplementary Data 11

Supplementary Data 12

Supplementary Data 13

Supplementary Data 14

Supplementary Data 15

Reporting Summary

## Data Availability

The datasets generated during the current study are available in the China National GeneBank DataBase (CNGBdb) (https://db.cngb.org/) with project accession CNP0001656, which includes Pacbio, Hi–C and transcriptome raw data. PacBio assembly accession number is CNA0050867. The Hi–C assembly accession number is CNA0050868. The accession number of RNA-seq data is CNX0296571 and the corresponding sample information is summarized in Supplementary Table 8. The numerical values used to generate the plots in Fig. 1d, e were provided as Supplementary Data 1. The source data underlying Fig. 1d were downloaded from NCBI. The source data underlying Fig. 1e and Fig. 7 are provided in Supplementary Data 2–4 and Supplementary Data 11, respectively.

## Code availability

The DEseq2 and WGCNA R code are available in Supplementary Data 14.
