# Supplementary material for: A draft genome of the medicinal plant Cremastra appendiculata (D. Don) provides insights into the colchicine biosynthetic pathway
Source: Commun Biol. 2022 Nov 25;5:1294. doi: 10.1038/s42003-022-04229-4 (PMC9700805; doi:10.1038/s42003-022-04229-4)
Supplement: Supplementary file 1 — Supplementary Information [file 42003_2022_4229_MOESM1_ESM.pdf]

Supplementary Information

A draft genome of the medicinal plant *Cremastra appendiculata* (D. Don) provides insights into colchicine biosynthetic pathway

Jing Wang [1,#], Jingjing Xie [2,3,#], Haixia Chen[4,5,#], Xia Qiu [2,3], Hai Cui [6], Yijiang Liu [6], Sunil Kumar Sahu [4], Dongming Fang [4], Tengyan Li [7], Mei Wang [4], Yewen Chen [4,5], Huan Liu [4,8,*], Jianyong Zhang [2,3,*], Binbin Wang [7,*]

1. Department of Medical Genetics and Developmental Biology, School of Basic Medical Sciences, Capital Medical University, Beijing, 10069, China.
2. Department of Rheumatism, Shenzhen Traditional Chinese Medicine Hospital, Shenzhen, Guangdong, China.
3. The Fourth Clinical Medical College of Guangzhou University of Chinese Medicine, Shenzhen, Guangdong, China.
4. State Key Laboratory of Agricultural Genomics, BGI-Shenzhen, Shenzhen 518120, China
5. College of Life Sciences，University of Chinese Academy of Sciences，Beijing 100049，China
6. School of Traditional Chinese Medicine, Capital Medical University, Beijing, 10069, China.
7. Center for Genetics, National Research Institute for Family Planning, Beijing, China.
8. Department of Biology, University of Copenhagen, Copenhagen, Denmark.

Corresponding author e-mail: Binbin Wang: wbbahu@163.com; Jianyong Zhang: 13823396319@163.com; Huan Liu: liuhuan@genomics.cn

Supplementary Tables

Supplementary Tables 1. Statistics of genome and gene Characteristic of the *C. appendiculata* genomes.

Protein-coding regions	count
Number of gene models *4	20,991
Gene density (genes per Mb)	8
Exonic proportion, including introns (bp)	242,398,077
Mean/Median gene size (bp)	11547.71/3563
Mean/Median CDS size (bp)	1386.10/1176
Number of exons	107,664
Number of bp included in exons	29,095,701
Mean/Median exon size (bp)	270.25/137
Mean number of exons per gene	5.13
Number of introns	86,673
Number of bp included in introns	213,302,375
Mean/Median intron size (bp)	2,461.00/275

Note: *1, For protein-coding, need to satisfy the conditions: aa > 30bp; non-pseudogene; keep only the longest transcript for one gene.

Supplementary Table 2. Non-coding RNA genes in the *C. appendiculata* genome.

Type		Copy (w)	Average length(bp)	Total length(bp)	% of genome
miRNA		97	125.8454	12,207.00	0.000517
tRNA		430	76.52093	32,904.00	0.001393
rRNA	rRNA	2,926	184.9149	541,061.00	0.022904
	18S	304	838.5197	254,910.00	0.010791
	28S	542	140.1494	75,961.00	0.003215
	5.8S	136	151.0735	20,546.00	0.00087
	5S	1,944	97.5535	189,644.00	0.008028
snRNA	snRNA	728	109.386	79,633.00	0.003371
	CD-box	382	105.2304	40,198.00	0.001702
	HACA-box	56	130.7143	7,320.00	0.00031
	splicing	290	110.7414	32,115.00	0.001359

Supplementary Table 3. Statistics of Gene Families in *C. appendiculata* and other genomes.

Species	Genes number	Genes in families	Unclustered genes	Family number	Unique families	Expanded families	Contracted families	Average genes per family
V. vinifera	26,083	19,601	6,482	12,672	662	141	117	1.55
P. equestris	18,296	16,519	1,777	11,855	157	322	110	1.39
A. shenzhenica	21,743	18,365	3,378	11,734	518	338	77	1.57
S. polyrhiza	20,295	12,276	8,019	9,939	201	23	335	1.24
A. comosus	27,024	22,020	5,004	12,964	738	344	71	1.7
D. catenatum	22,260	20,239	2,021	12,767	189	476	19	1.59
P. dactylifera	25,205	22,751	2,454	12,590	242	278	33	1.81
O. sativa	42,069	30,885	11,184	17,106	1,328	1,845	85	1.81
A. trichopoda	26,846	19,316	7,530	12,397	1,063	72	123	1.56
A. officinalis	24,893	22,131	2,762	11,450	514	272	136	1.93
B. distachyon	34,307	26,212	8,095	16,095	679	1,093	112	1.63
M. acuminate	35,862	25,277	10,585	12,575	533	482	60	2.01
A. thaliana	27,130	23,366	3,764	12,672	716	197	129	1.84
C. appendiculata	20,991	20,551	440	12,146	94	489	54	1.69
S. bicolor	34,124	26,828	7,296	16,390	768	1,215	58	1.64
P. trichocarpa	41,296	33,681	7,615	14,216	1,144	593	19	2.37

Supplementary Table 4. Statistics of Syntenic Blocks between *C. appendiculata* and *P. equestris* genome.

A vs B	of_Syntenic_Blocks (Scaffold)	Average_Syntenic_Gene _Pairs_Per_Block	of_Syntenic_Gene_Pairs	Mean_Block_Length
C. appendiculata_C. appendiculata	61	11.7541	717	48955.4344
C. appendiculata_P. equestris	415	8.3711	3474	539692.7687/313800.7590
P. equestris_P. equestris	25	6.96	174	899760.6

Supplementary Table 5. Ks distribution related to speciation events between *C. appendiculata* and *P. equestris* genome.

Type	C. appendiculata_P. equestris
KS peak	0.3462
Variance	0.0007
SD	0.0278
Time (Ma)	39.33 +/- 3.16

Supplementary Table 6. The candidate gene list of colchicine pathway in *C. appendiculata* genome.

Name	ID
Ca4CL	CAPP02019<br>CAPP03360<br>CAPP09368
CaAER	CAPP02238<br>CAPP14911
CaCYP76AD5	CAPP14828
CaCCR	CAPP06280<br>CAPP21000
CaCYP71FB1	CAPP10396<br>CAPP13186<br>CAPP18159<br>CAPP18160<br>CAPP18161
CaC4H	CAPP11424<br>CAPP14458
CaC4H	CAPP19256
CaCYP75A109/CYP75A110	CAPP21024<br>CAPP17179<br>CAPP17180
CaDAHPS	CAPP06476
CaNMT	CAPP08794
CaOMT1-3	CAPP21050<br>CAPP07203<br>CAPP15509<br>CAPP20807
CaOMT4	CAPP12144<br>CAPP12145<br>CAPP16415
CaPAL	CAPP01280<br>CAPP03261<br>CAPP03997<br>CAPP10338<br>CAPP17355<br>CAPP19837
CaDDC	CAPP17307

Supplementary Table 7. The mapping rate of RNA reads to *C. appendiculata* genome using both Hisat2 and Tophat2.

Sample	HISAT2	Tophat2
P2.1	55.42%	53.30%
P2.3	90.56%	86.00%
S2.1	87.93%	85.20%
S2.3	55.79%	53.90%
L2.3	58.93%	57.50%
L2.2	58.11%	56.40%
P4.2	86.01%	82.10%
P4.3	89.90%	84.40%
S4.1	90.84%	85.70%
S4.2	92.60%	87.40%
S4.3	92.10%	87.60%
L4.2	91.69%	86.60%
L4.3	92.62%	87.90%
P6.1	90.32%	85.40%
P6.3	85.37%	81.10%
S6.1	91.49%	88.00%
S6.2	91.29%	86.30%
S6.3	91.34%	86.20%
L6.1	92.24%	87.00%
L6.2	90.85%	85.40%
L6.3	90.31%	84.60%

Note: "P": Pseudobulb, "S": Stem, "L": Leaf. 2: Two-years old, 4: Four-years, 6: Six-years. ".1, .2, .3" means sample duplications.

Supplementary Table 8. The sample information of RNA-seq data.

Run ID	Barcode	sample
CNR0366392	589	biennials,leaf1
CNR0366385	540	biennials,leaf2
CNR0366388	543	biennials,pseudobulb1
CNR0366387	542	biennials,pseudobulb2
CNR0366400	595	biennials,stem1
CNR0366396	590	biennials,stem2
CNR0366380	545	four-years,leaf1
CNR0366391	579	four-years,leaf2
CNR0366390	577	four-years,pseudobulb1
CNR0366386	541	four-years,pseudobulb2
CNR0366394	578	four-years,stem1
CNR0366381	546	four-years,stem2
CNR0366389	544	four-years,stem3
CNR0366382	547	six-years,leaf1
CNR0366398	593	six-years,leaf2
CNR0366384	557	six-years,leaf3
CNR0366399	594	six-years,pseudobulb1
CNR0366395	580	six-years,pseudobulb2
CNR0366393	591	six-years,stem1
CNR0366383	548	six-years,stem2
CNR0366397	592	six-years,stem3

Supplementary Figures

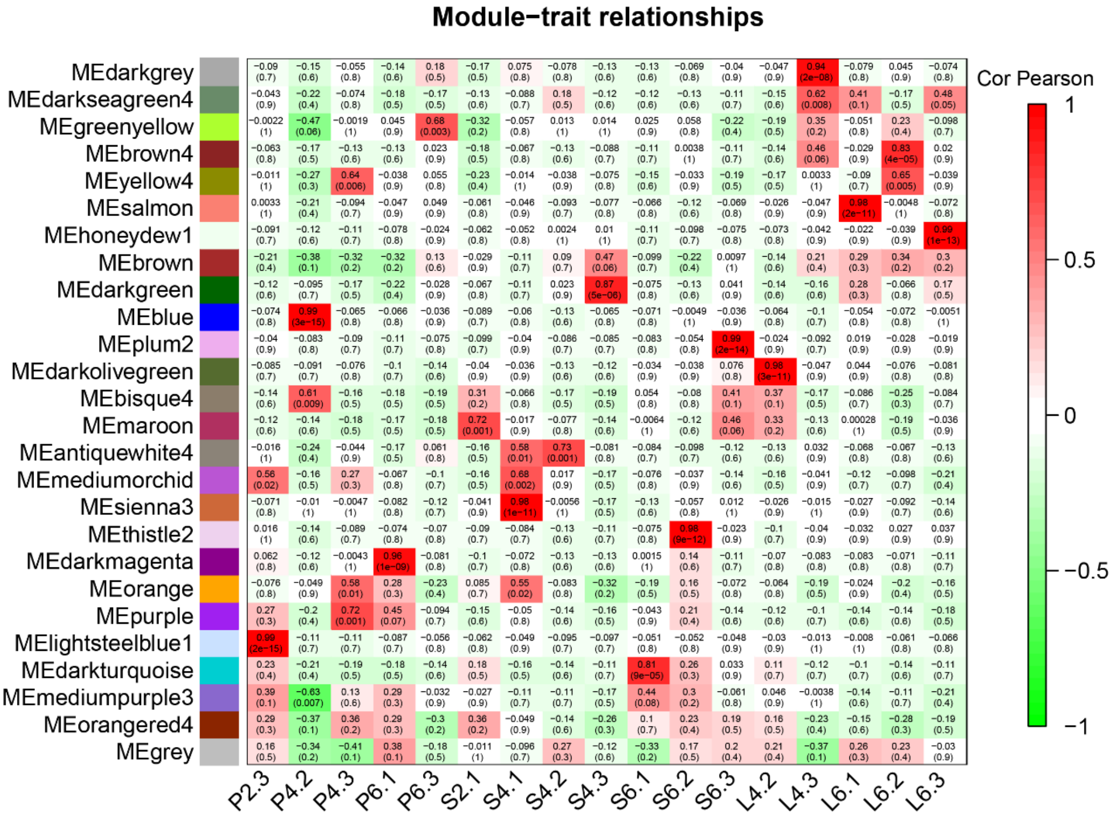

Supplementary Figure 1. The heatmap about the relationships between modules and plant tissues. The Y-axis represents kinds of modules in different colors. The X-axis represents different tissues. Red and green blocks indicate the correlation coefficients. “P”: Pseudobulb, “S”: Stem, “L”: Leaf. 2: biennialstwo-years old, 4: Four-years, 6: Six-years. “.1, .2, .3” means sample duplications. Cor Pearson means calculated the correlation between modules and sample traits by pearson method.

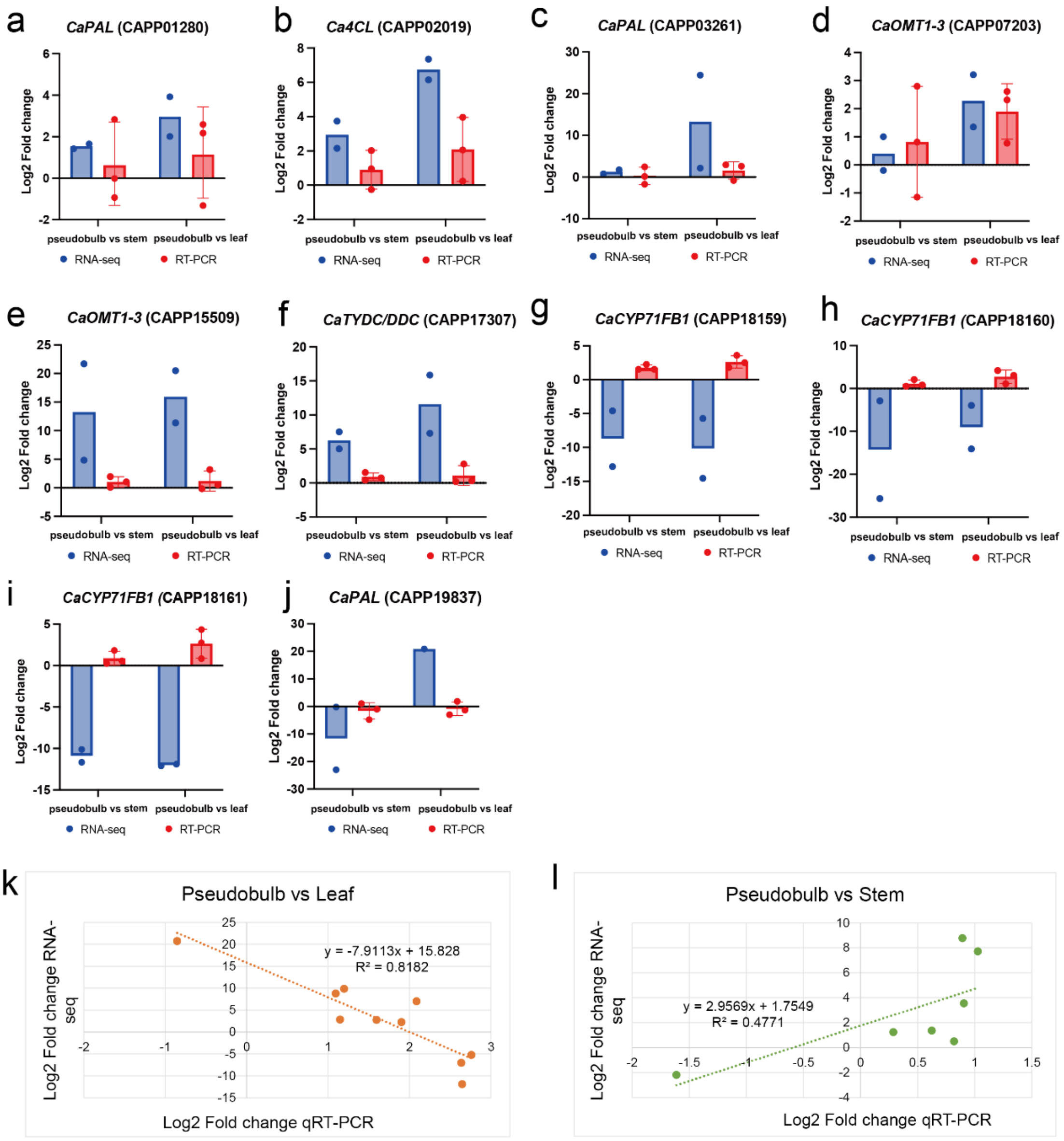

Supplementary Figure 2. Validation of differentially expressed genes (DEGs) and correlation of gene expression. a-j. Validation of ten DEGs by qRT-PCR. The x-axis represents the groups of different expression analysis. The y-axis indicates the log2 fold change values. Blue and red bars represent RNA-seq and RT-PCR data, respectively. All comparisons are relative to pseudobulb and CAPP06738 was selected as reference gene. k-l. The comparison of RT-PCR and RNA-seq data. The Pearson correlation coefficient was calculated by log2 fold change values of RNA-seq and qRT-PCR data. Error bars are ± SE, n=2-3.

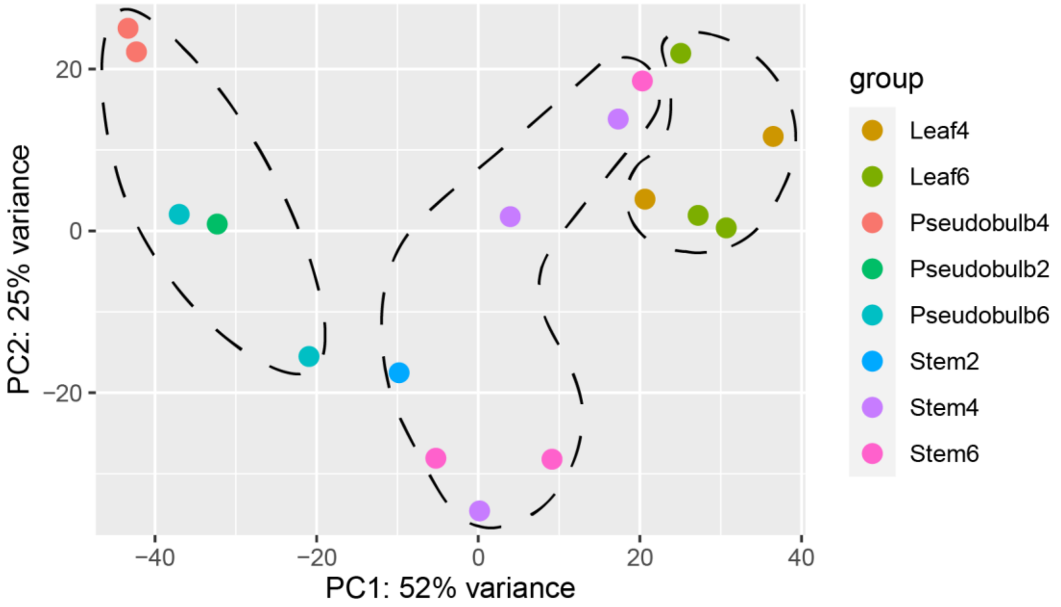

Supplementary Figure 3. The PCA results of the RNA-seq data. The number 2,4,6 means two-years old, four-years and six-years samples, respectively.
