# Supplementary material for: A draft genome of the medicinal plant Cremastra appendiculata (D. Don) provides insights into the colchicine biosynthetic pathway
Source: Commun Biol. 2022 Nov 25;5:1294. doi: 10.1038/s42003-022-04229-4 (PMC9700805; doi:10.1038/s42003-022-04229-4)
Supplement: Supplementary file 2 — Description of Additional Supplementary Files [file 42003_2022_4229_MOESM2_ESM.pdf]

Description of Additional Supplementary Files

File name: Supplementary Data1

Description: The numerical values used to generate the plots in Figure 1d-e.

File name: Supplementary Data 2

Description: The source data of Apostasia shenzhenica behind Figure 1e.

File name: Supplementary Data 3

Description: The source data of Dendrobium catenatum behind Figure 1e.

File name: Supplementary Data 4

Description: The source data of Phalaenopsis equestris behind Figure 1e.

File name: Supplementary Data 5

Description: Repeat elements annotated in this genome.

File name: Supplementary Data 6

Description: Functional enrichment analysis of expansion families of C. appendiculata genome.

File name: Supplementary Data 7

Description: Functional enrichment analysis of unique families of C. appendiculata genome.

File name: Supplementary Data 8

Description: KEGG enrichment analysis of whole genome duplication (WGD) genes of C. appendiculata genome.

File name: Supplementary Data 9

Description: Gene trees of each candidate colchicine gene family identified in C. appendiculata genome.

File name: Supplementary Data 10

Description: GO enrichment analysis of up- and down-regulated DEGs between pseudobulb vs leaf and pseudobulb vs stem, respectively.

File name: Supplementary Data 11

Description: KEGG enrichment analysis of up- and down-regulated DEGs between pseudobulb vs leaf and pseudobulb vs stem, respectively.

File name: Supplementary Data 12

Description: The original CT values and related data of qRT-PCR analysis.

File name: Supplementary Data 13

Description: Transcription factors (TFs), transcription regulators (TRs), protein kinases (PKs), Carbohydrate-Active Enzymes, Resistance genes (CAZymes) and KofamKOALA annotation result in this genome.

File name: Supplementary Data 14

Description: The R script of DEseq2 and WGCNA used in this paper.

File name: Supplementary Data 15

Description: The primer list of genes identified by qRT-PCR.
