# Supplementary figures and images for: A draft genome of the medicinal plant Cremastra appendiculata (D. Don) provides insights into the colchicine biosynthetic pathway
Source: Commun Biol. 2022 Nov 25;5:1294. doi: 10.1038/s42003-022-04229-4 (PMC9700805; doi:10.1038/s42003-022-04229-4)

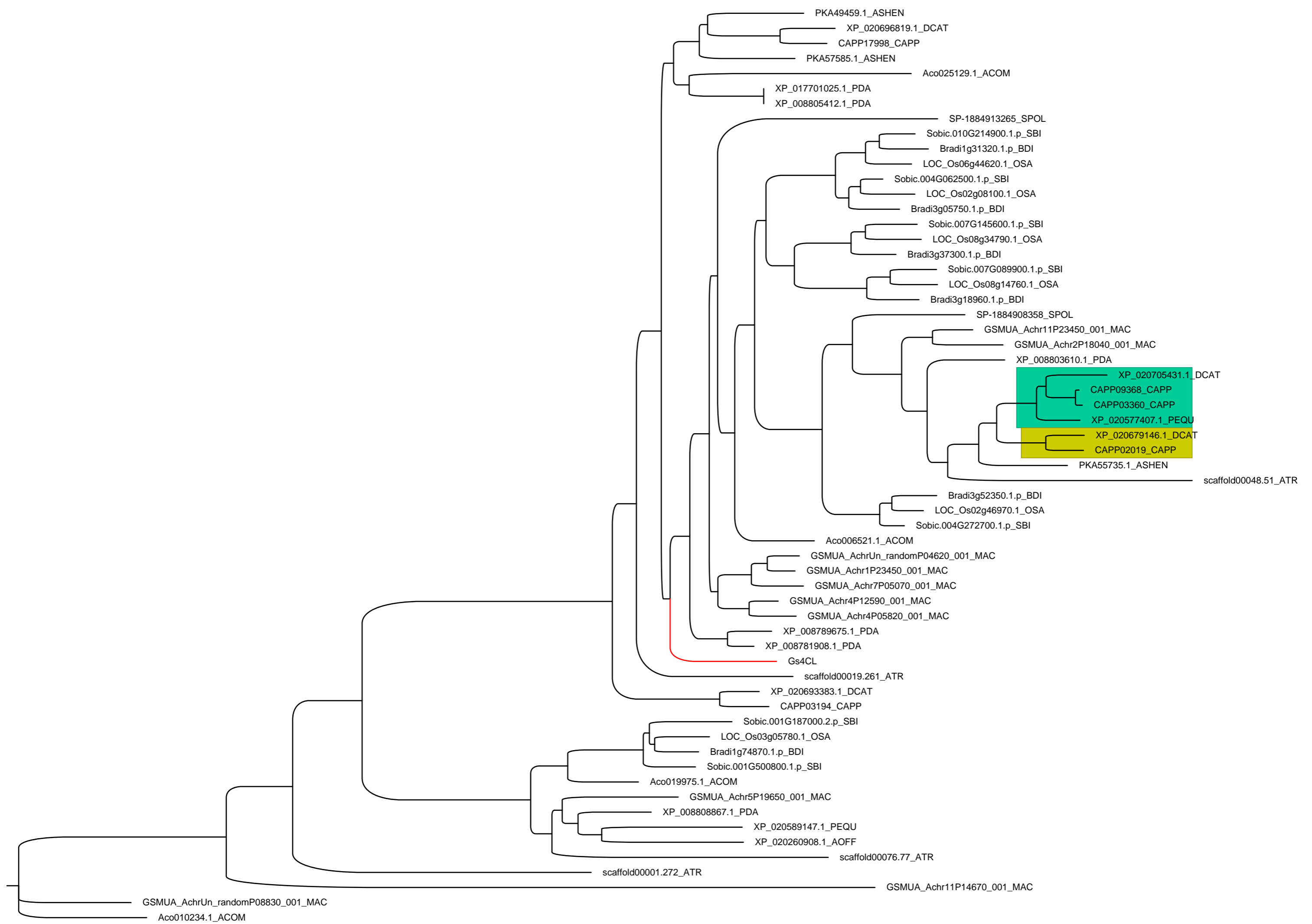

0.2

Supplement: Supplementary file 11 — Supplementary Data 9 [file 42003_2022_4229_MOESM11_ESM.zip › Ca4CL.tree.newick.pdf]

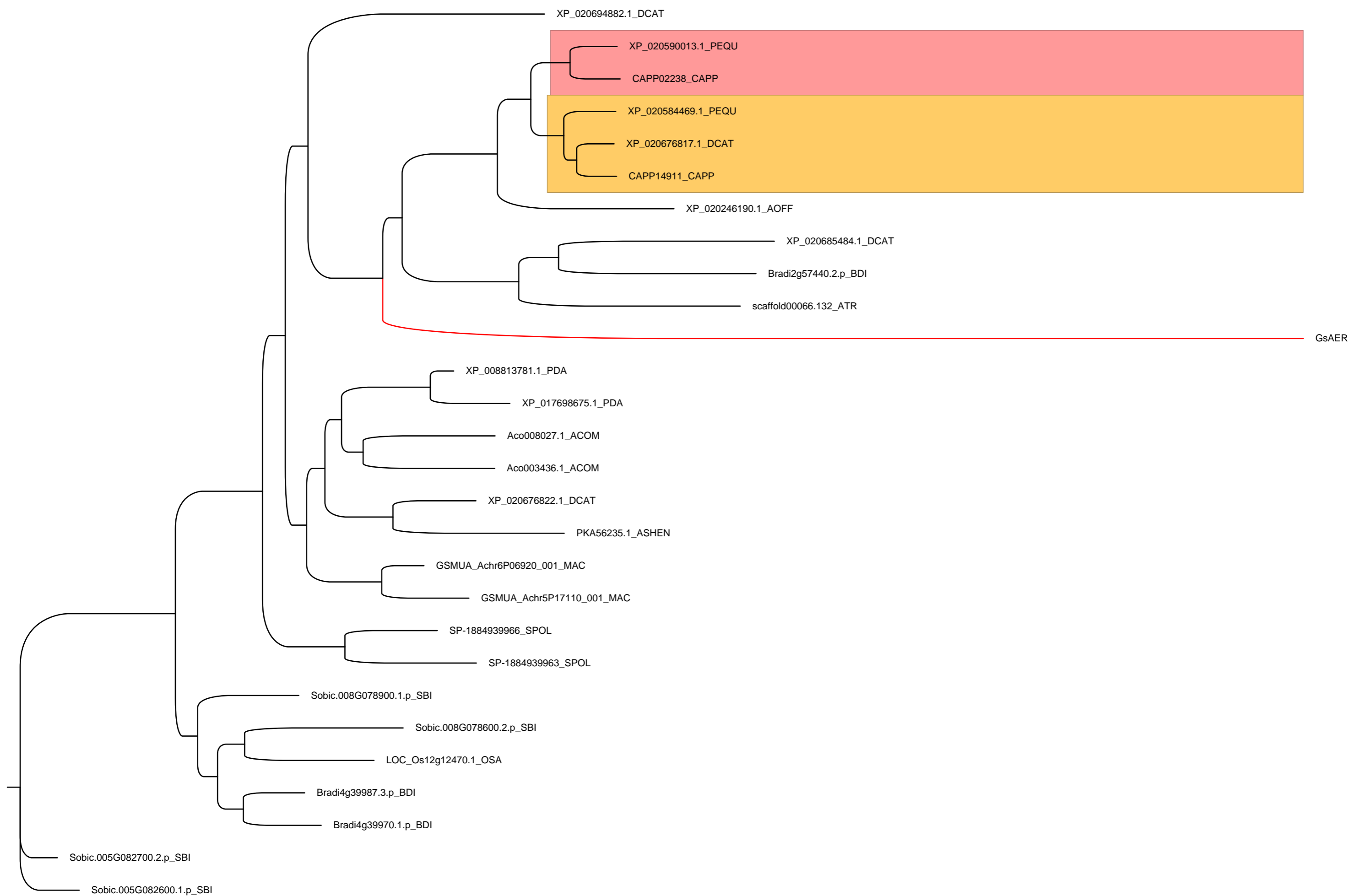

0.2

Supplement: Supplementary file 11 — Supplementary Data 9 [file 42003_2022_4229_MOESM11_ESM.zip › CaAER.tree.newick.pdf]

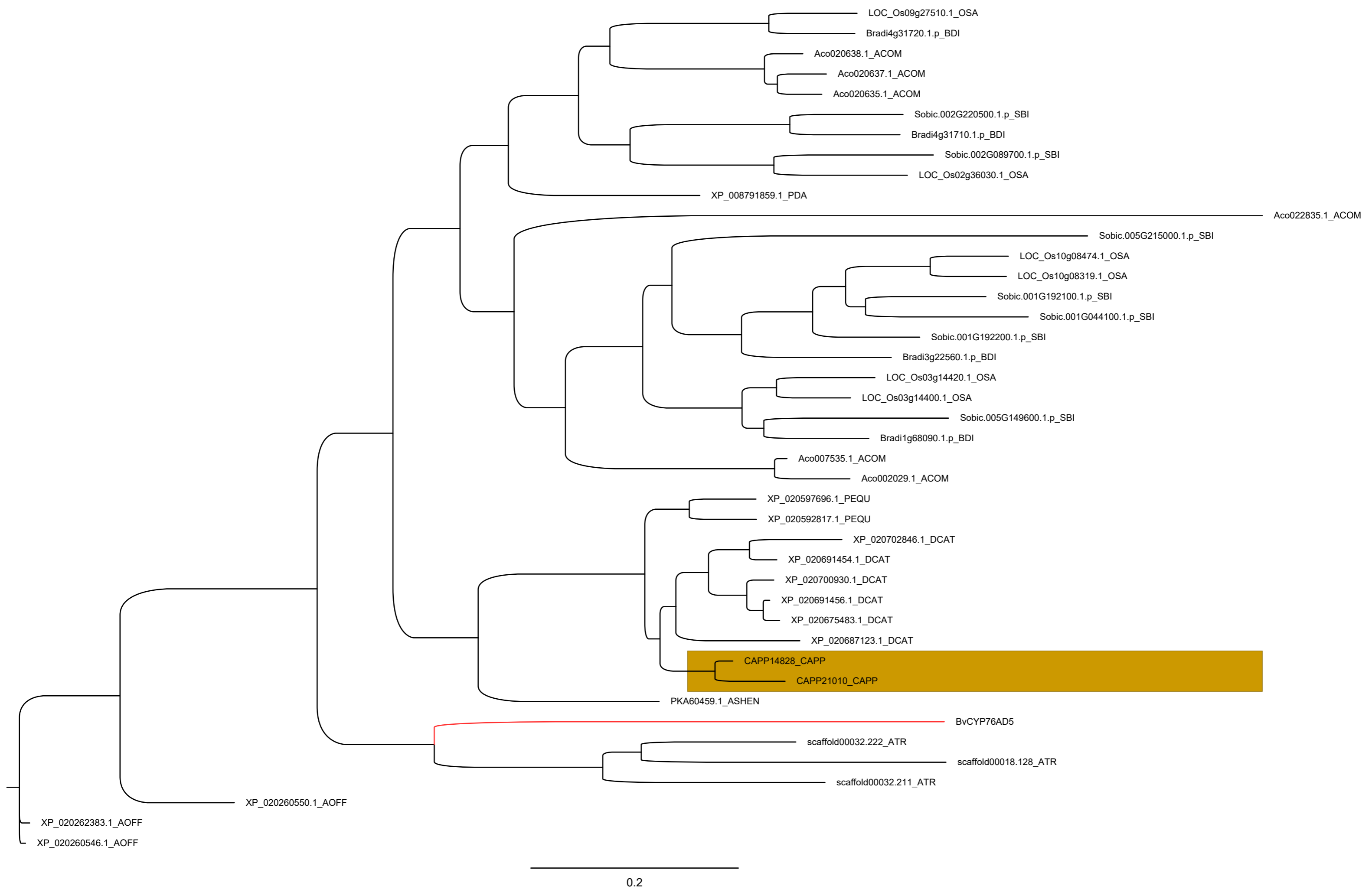

Supplement: Supplementary file 11 — Supplementary Data 9 [file 42003_2022_4229_MOESM11_ESM.zip › CaBvCYP76AD5.tree.newick.pdf]

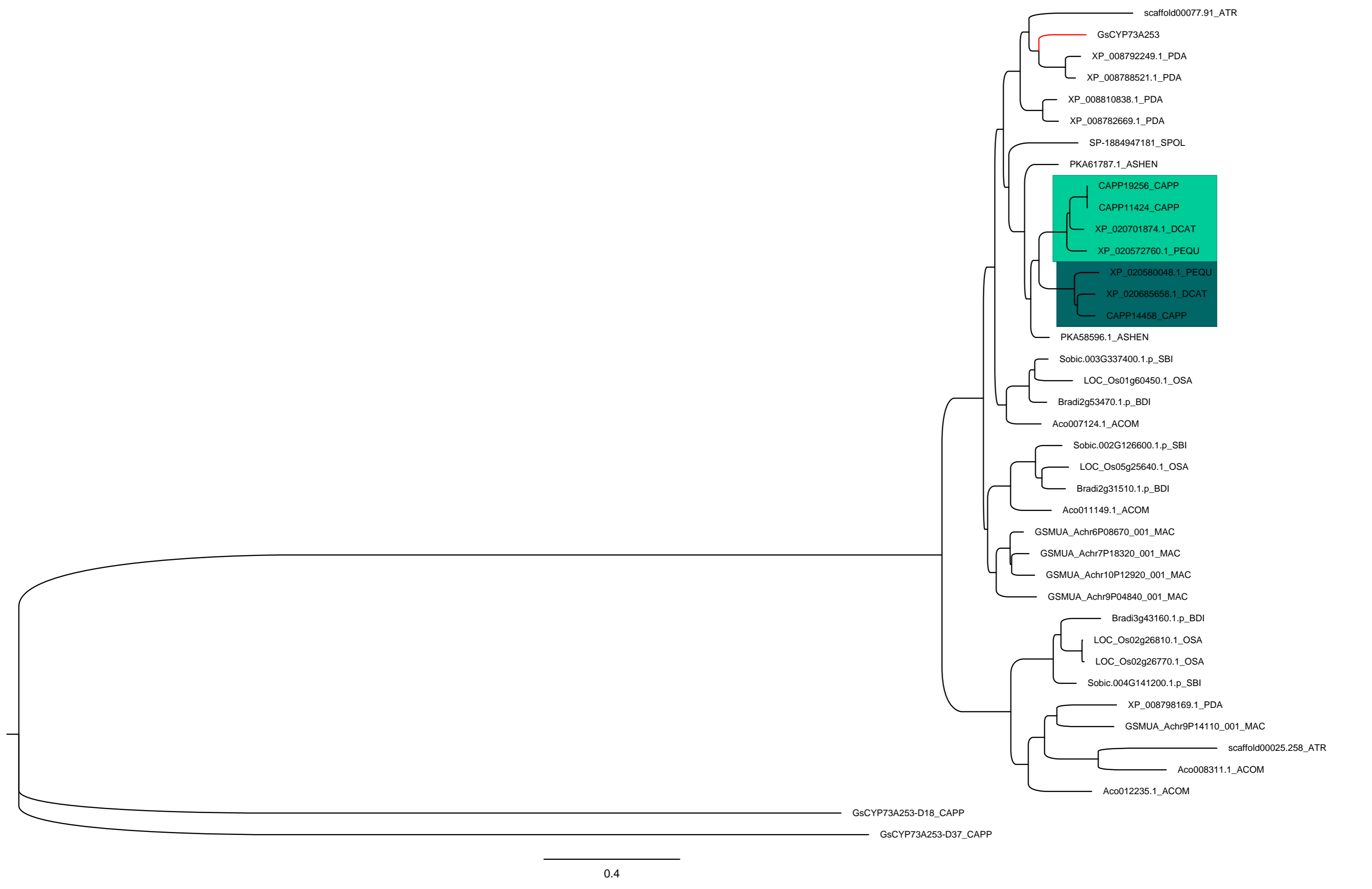

Supplement: Supplementary file 11 — Supplementary Data 9 [file 42003_2022_4229_MOESM11_ESM.zip › CaC4H.tree.newick.pdf]

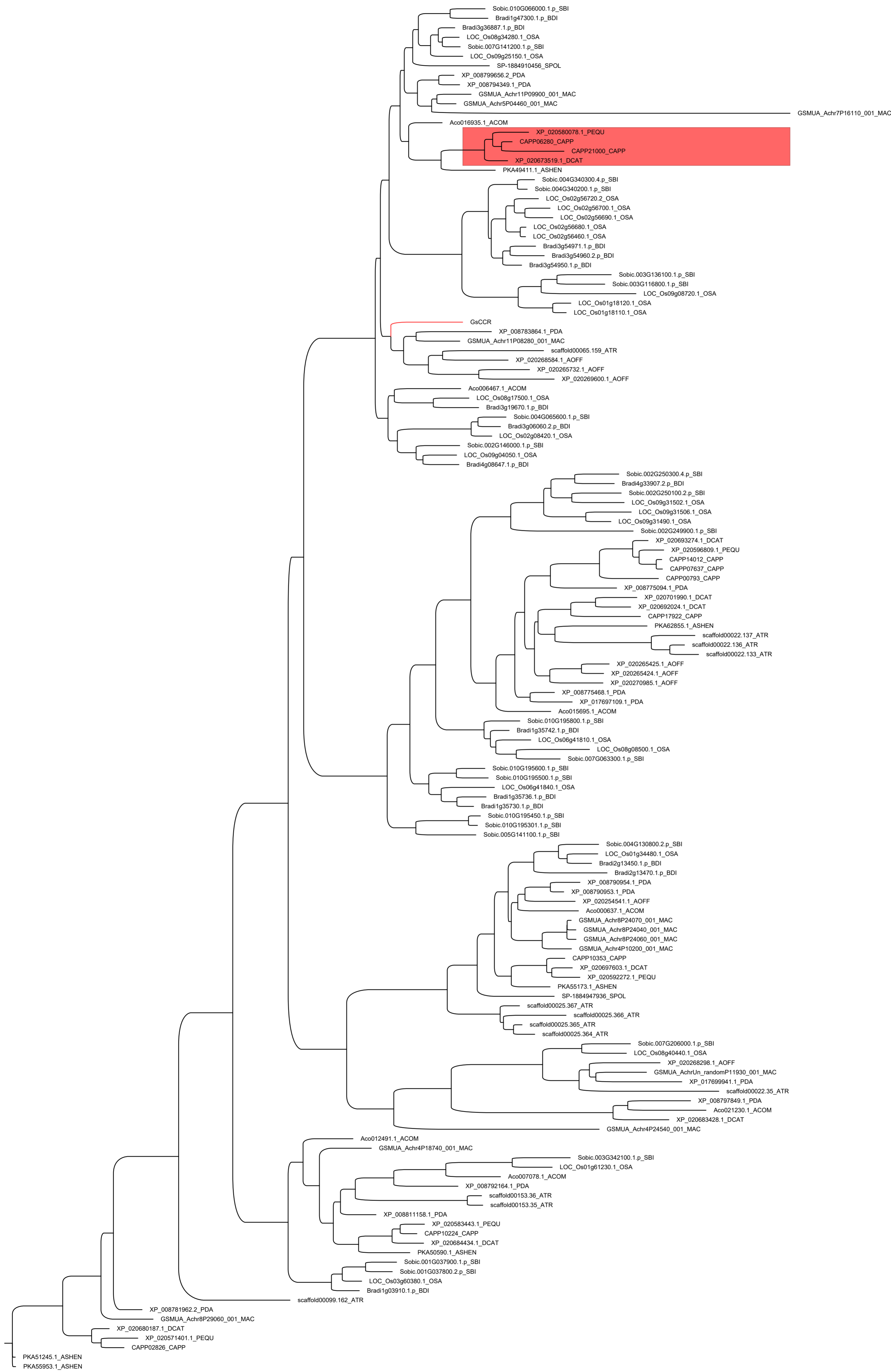

0.2

Supplement: Supplementary file 11 — Supplementary Data 9 [file 42003_2022_4229_MOESM11_ESM.zip › CaCCR.tree.newick.pdf]

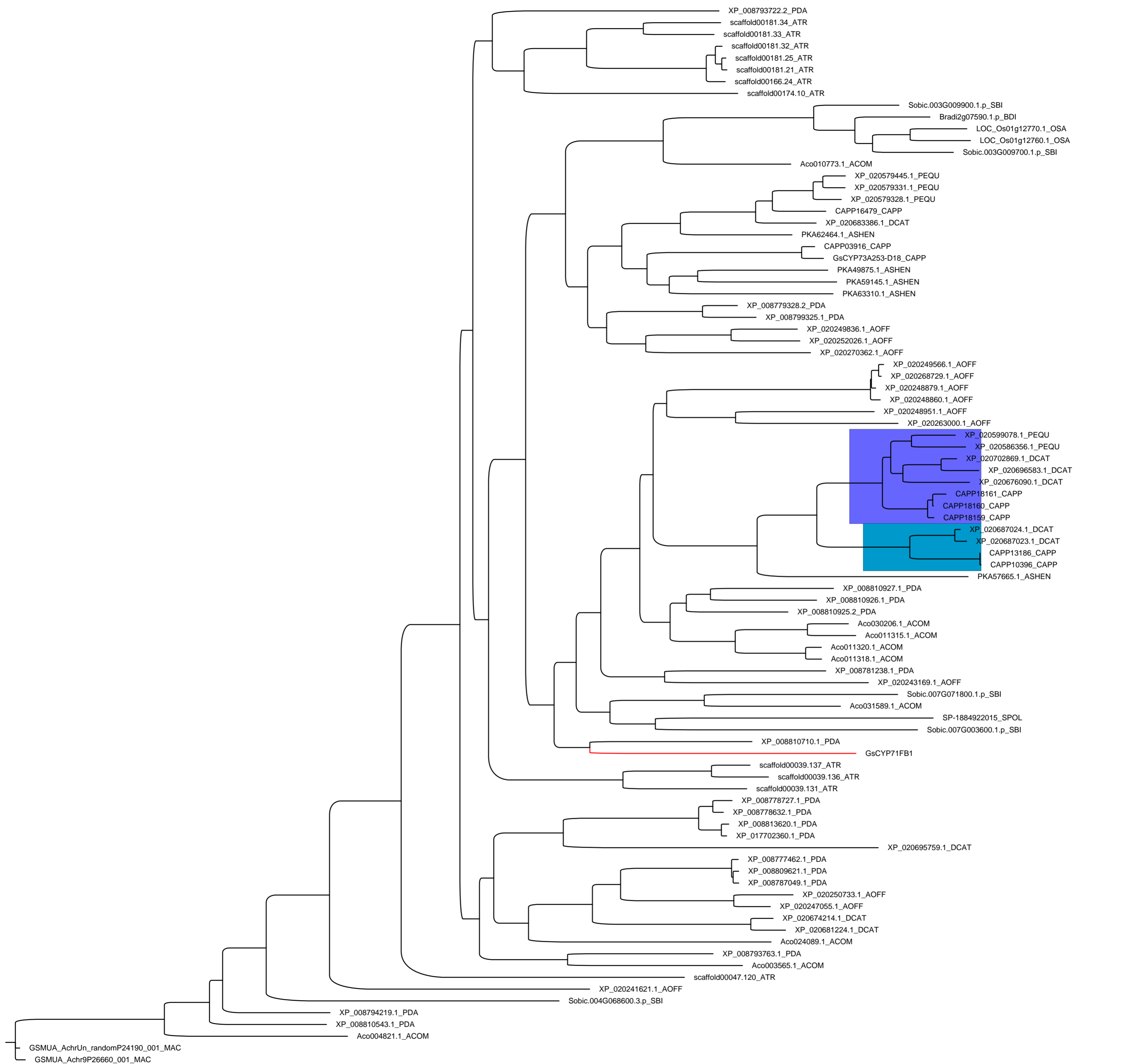

0.2

Supplement: Supplementary file 11 — Supplementary Data 9 [file 42003_2022_4229_MOESM11_ESM.zip › CaCYP71FB1.tree.newick.pdf]

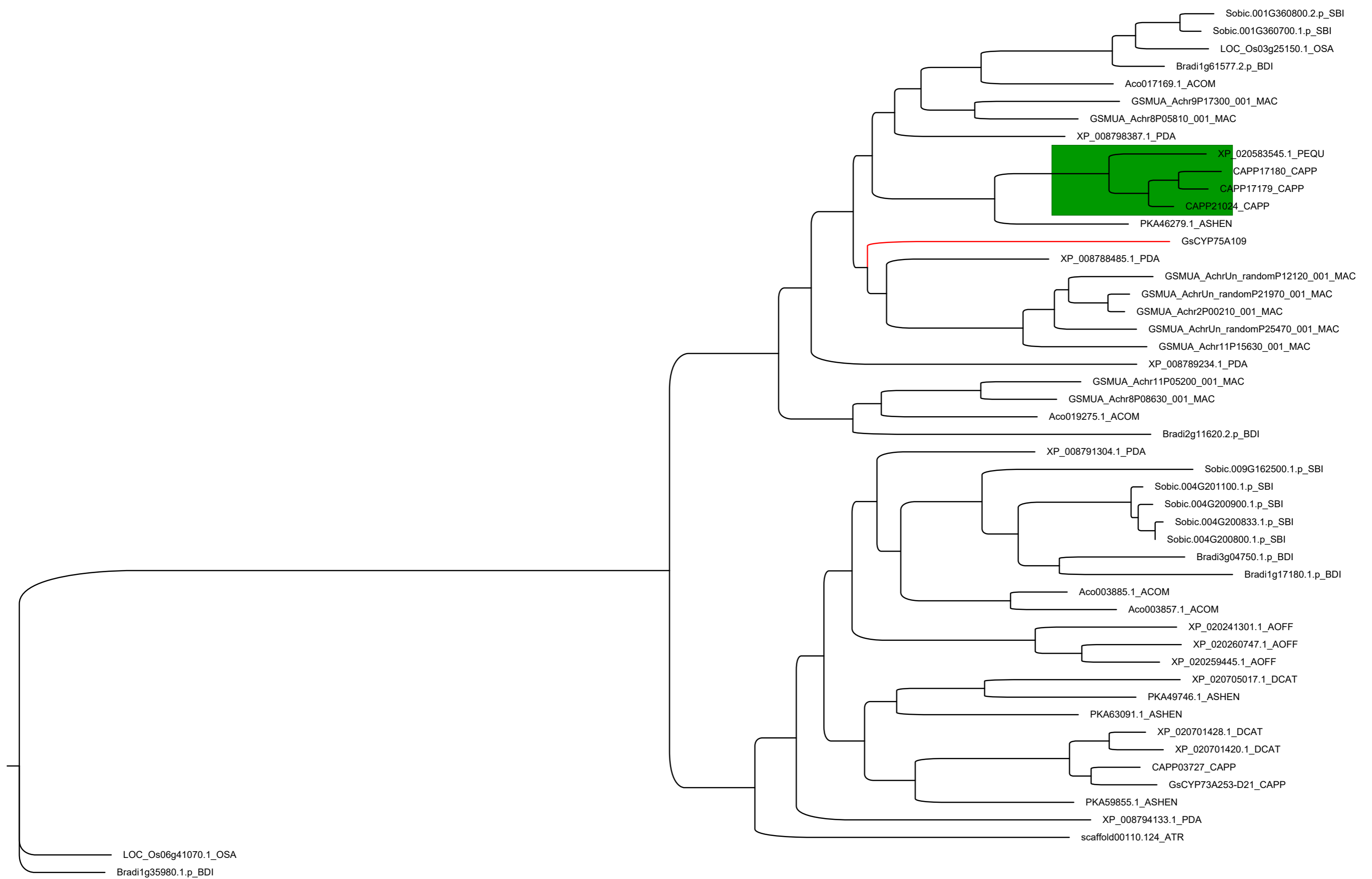

0.2

Supplement: Supplementary file 11 — Supplementary Data 9 [file 42003_2022_4229_MOESM11_ESM.zip › CaCYP75A109.tree.newick.pdf]

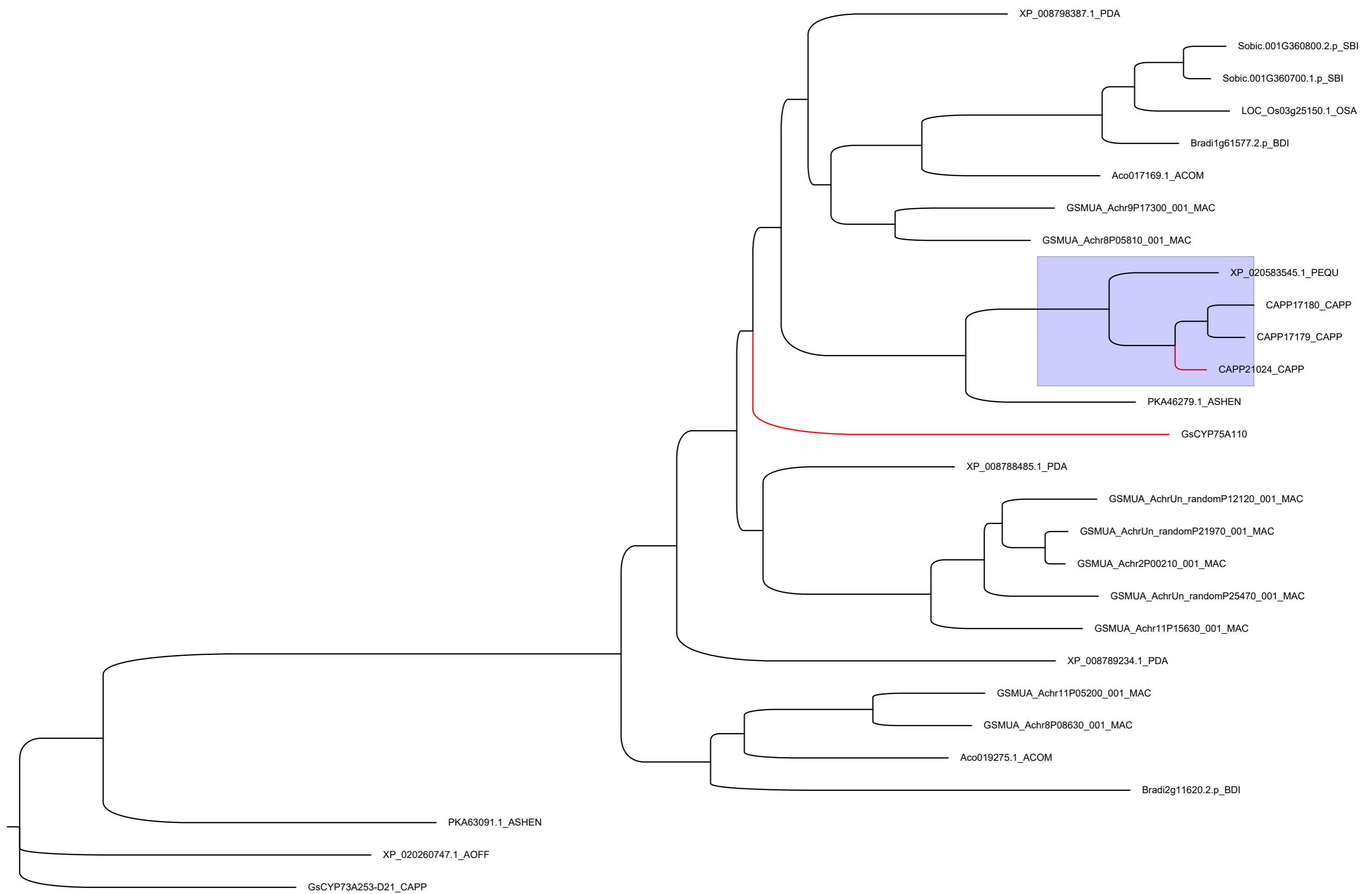

0.2

Supplement: Supplementary file 11 — Supplementary Data 9 [file 42003_2022_4229_MOESM11_ESM.zip › CaCYP75A110.tree.newick.pdf]

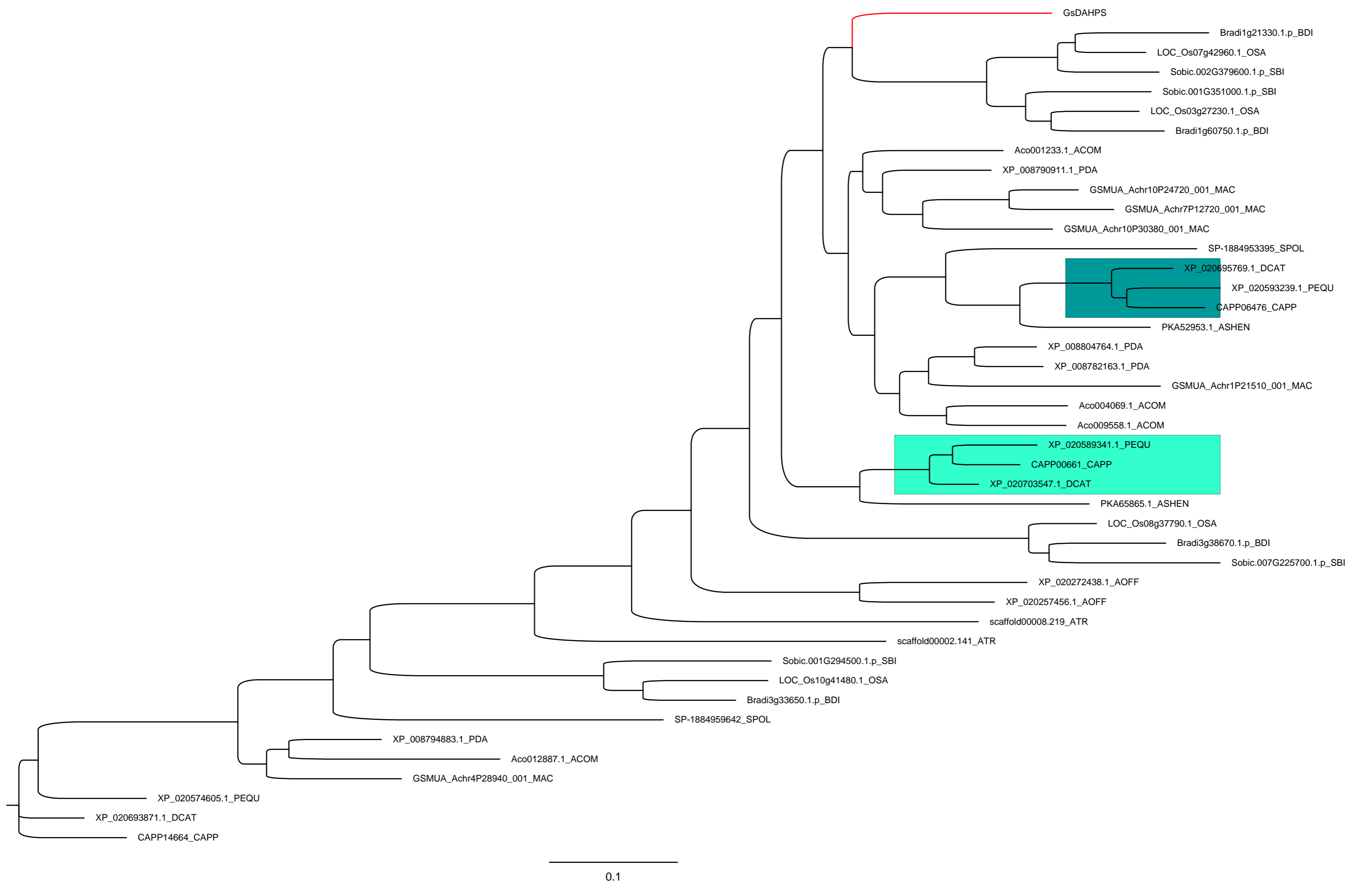

Supplement: Supplementary file 11 — Supplementary Data 9 [file 42003_2022_4229_MOESM11_ESM.zip › CaDAHPS.tree.newick.pdf]

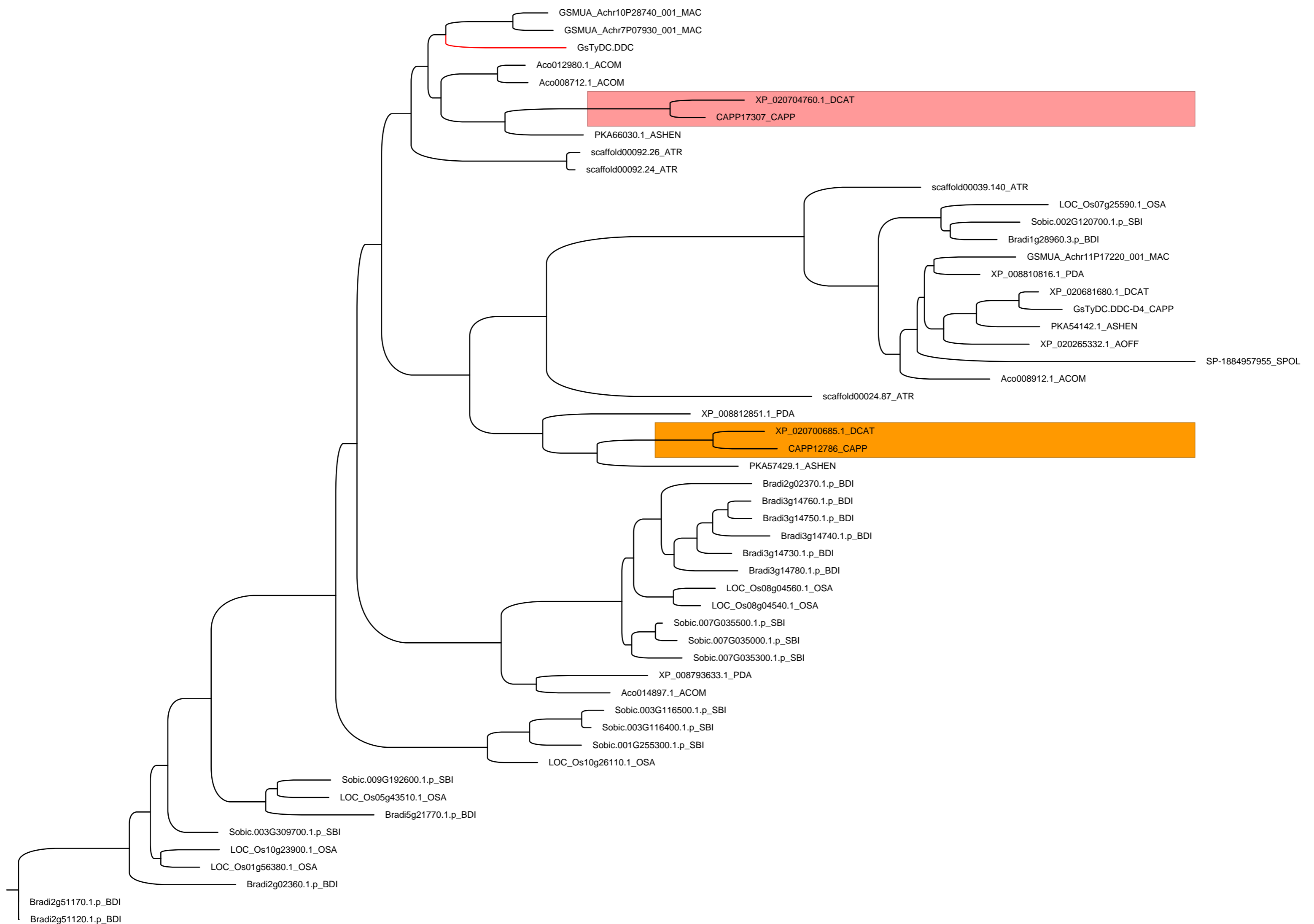

0.2

Supplement: Supplementary file 11 — Supplementary Data 9 [file 42003_2022_4229_MOESM11_ESM.zip › CaDDC.tree.newick.pdf]

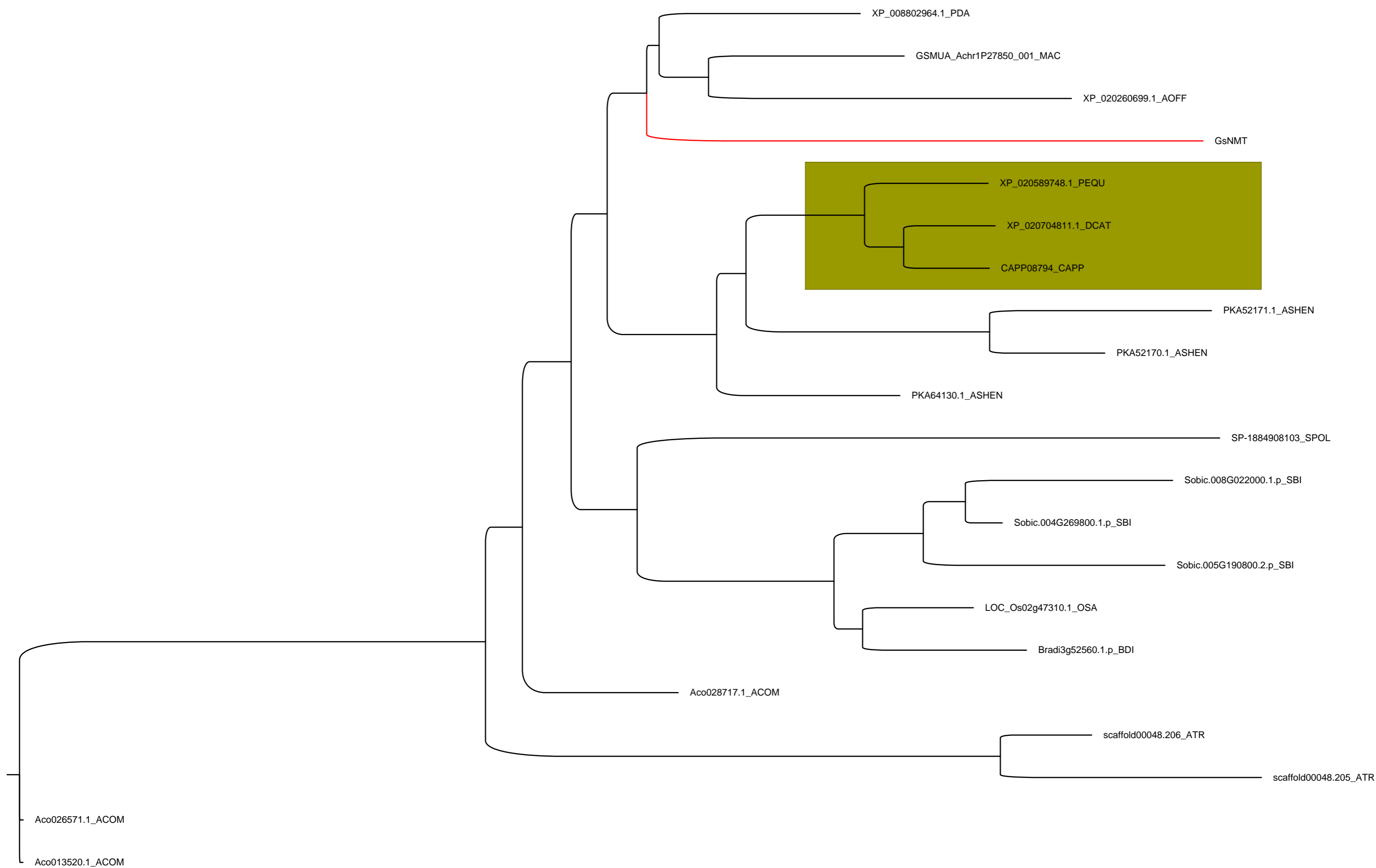

0.08

Supplement: Supplementary file 11 — Supplementary Data 9 [file 42003_2022_4229_MOESM11_ESM.zip › CaNMT.tree.newick.pdf]

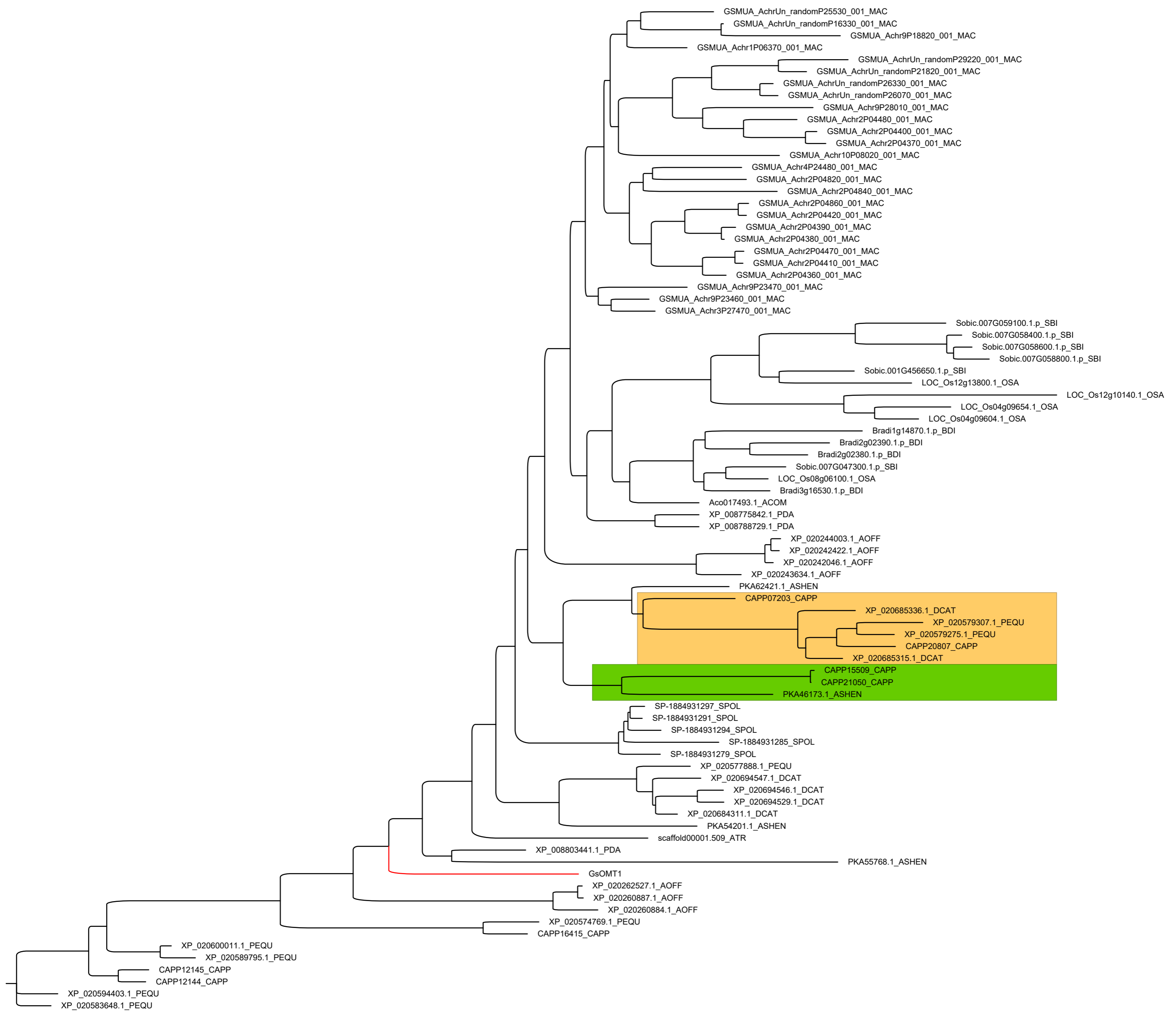

0.2

Supplement: Supplementary file 11 — Supplementary Data 9 [file 42003_2022_4229_MOESM11_ESM.zip › CaOMT1-3.tree.newick.pdf]

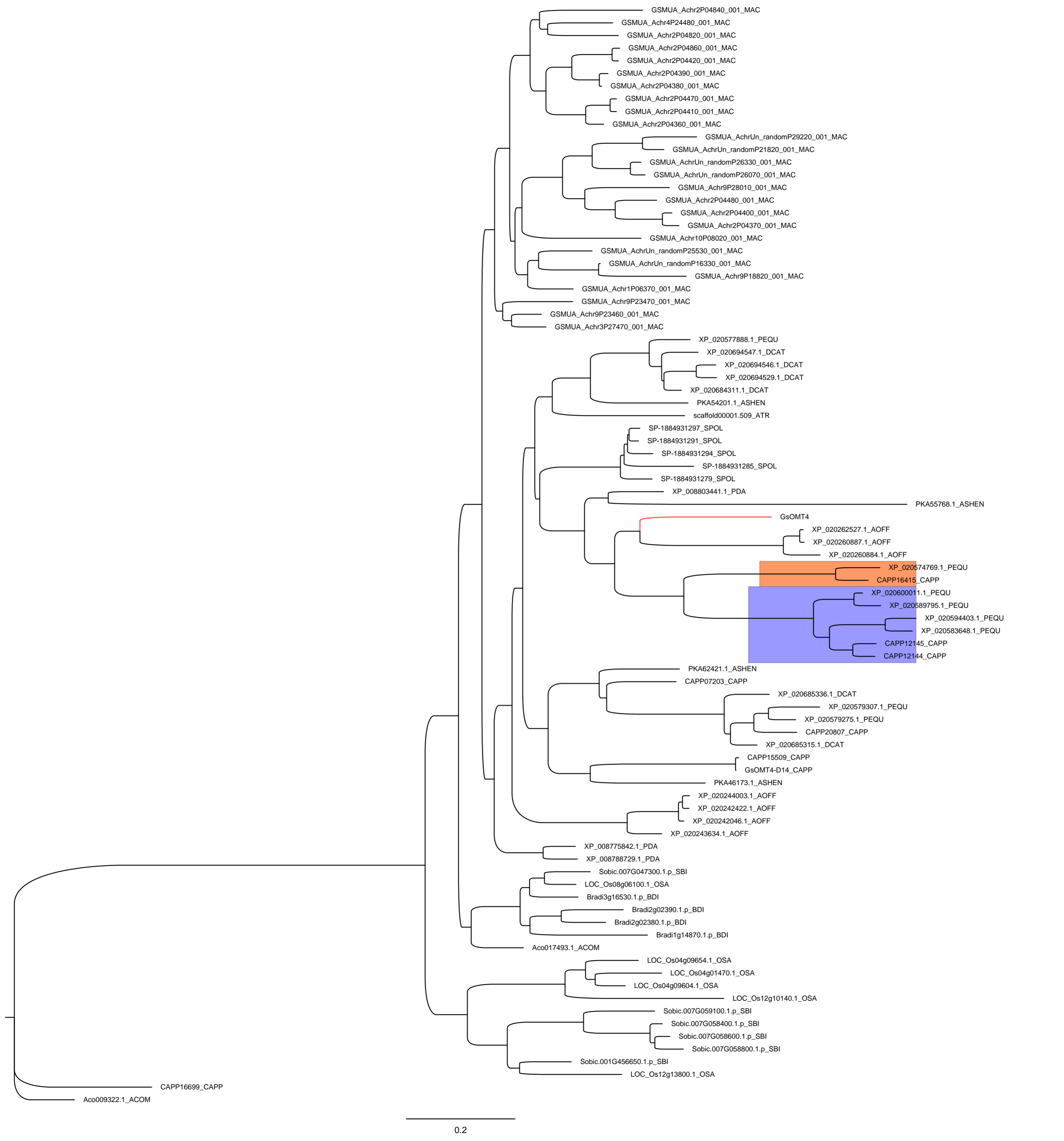

Supplement: Supplementary file 11 — Supplementary Data 9 [file 42003_2022_4229_MOESM11_ESM.zip › CaOMT4.tree.newick.pdf]

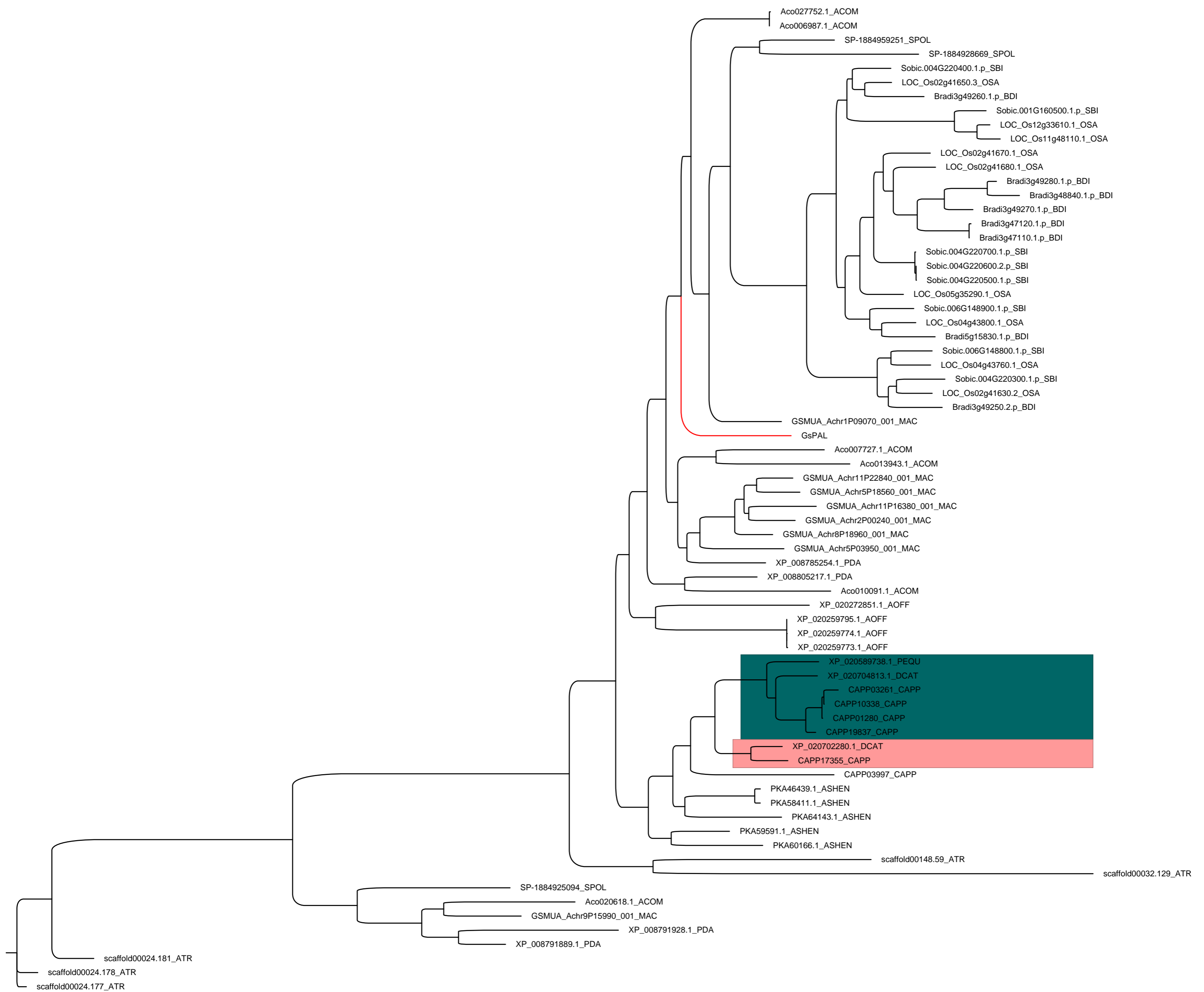

0.2

Supplement: Supplementary file 11 — Supplementary Data 9 [file 42003_2022_4229_MOESM11_ESM.zip › CaPAL.tree.newick.pdf]
